# Whole-genome sequencing-based typing methods for *Clostridium butyricum* strains from clinical, animal, plant, and environmental sources

**DOI:** 10.1128/spectrum.02619-25

**Published:** 2025-12-12

**Authors:** Nadim Cassir, Victoria Mesa, Laurent Ferraris, Johanne Delannoy, Yousra Mikrat, Anthony Adjamian, Laure Diancourt, Christelle Mazuet, Frédéric Barbut, Bernard La Scola, Julio Aires

**Affiliations:** 1 Université Paris Cité, Inserm, U1139, FPRM, Paris, France; 2 IHU Méditerranée Infection 290815, Marseille, France; 3 Microbes Evolution Phylogeny and Infection (MEPHI), Aix-Marseille Université 128791, Marseille, France; 4 Assistance Publique-Hôpitaux de Marseille (AP-HM) 36900 , Marseille, France; 5 Institut Pasteur, Centre National de Référence des Bactéries anaérobies et Botulisme, Paris, France; Assistance Publique - Hopitaux de Paris Universite Paris Saclay, Clamart, France

**Keywords:** *Clostridium butyricum*, necrotizing enterocolitis, genotyping, wgMLST, cgMLST, cgSNP, chewBBACA, Snippy

## Abstract

*Clostridium butyricum* exhibits a dual role, acting not only as a probiotic but also as an opportunistic pathogen associated with neonatal necrotizing enterocolitis (NEC) and infant botulism. We aimed to establish high-resolution genotyping frameworks to improve molecular surveillance and outbreak investigations. We analyzed 297 *C. butyricum* genomes, including 200 isolates from preterm neonates across 13 French neonatal intensive care units over a 20-year period and 97 publicly available genomes. A core-genome multilocus sequence typing (cgMLST) scheme was developed using chewBBACA, defining 2,621 loci, and applied to genomes with ≥95% locus presence. Core-genome single-nucleotide polymorphism (cgSNP) analysis was performed for complementary resolution. Phylogenetic cgMLST classified isolates into nine major clades. Some clinical strains displayed clonal relationships, whereas others were geographically and temporally unrelated. All botulinum neurotoxin type E-producing strains were grouped within a single clade. NEC-associated isolates showed geographic and temporal clustering, but no clade was uniquely linked to NEC. cgSNP analysis identified 11 clusters with overall discriminatory power similar to cgMLST while providing finer resolution for NEC-related strains. We propose robust cgMLST and cgSNP schemes for *C. butyricum*, enabling high-resolution genotyping and supporting epidemiological surveillance and outbreak investigation of this emerging opportunistic pathogen in neonatal settings.

**IMPORTANCE:**

*Clostridium butyricum* has been identified in fecal samples from both asymptomatic neonates and cases of necrotizing enterocolitis (NEC). Using a large collection of strains from different origins and spatiotemporal contexts, we developed and established a cgMLST scheme for the molecular typing of *C. butyricum*. Our results show that most *C. butyricum* strains cluster independently of origin and spatiotemporal context factors. However, specific cgMLST clades of *C. butyricum* were found for plant and botulinum neurotoxin type E strains. Clonal strains were also identified. No specific cgMLST clade was found to be genetically associated with NEC. cgSNP showed higher discriminatory power compared to cgMLST. Importantly, cgSNP provided better discriminatory power for strain relatedness with respect to strains isolated from NEC patients.

## INTRODUCTION

*Clostridium butyricum*, first isolated from pig intestine by Prazmowski in 1880, belongs to cluster I *sensu stricto* and is the type species of the genus *Clostridium* (1). This anaerobic bacterium is recovered from human and animal feces (1) and from diverse environmental sources, with detection rates exceeding 30% in some surveys (2). In addition to its industrial use for producing organic acids, solvents, and hydrogen (3), *C. butyricum* is a gut commensal in healthy individuals but has also been associated with infant botulism and necrotizing enterocolitis (NEC) in preterm neonates (4). Some strains are marketed as probiotics with reported health benefits in humans and animals, yet probiotic-associated *C. butyricum* bacteremia has been documented (5). Furthermore, certain isolates carry transferable antibiotic resistance genes (6), underscoring the need for detailed molecular characterization.

Despite its clinical and industrial relevance, the genomic diversity of *C. butyricum* remains underexplored, with existing studies based on a limited number of strains (7). To our knowledge, no standardized whole-genome-based bacterial typing scheme exists for this species. For other pathogens, core-genome multilocus sequence typing (cgMLST) and core-genome single-nucleotide polymorphism (cgSNP) analyses are widely applied for epidemiological surveillance and source attribution (8–11). cgMLST, which assigns allelic profiles to the core genome, offers high reproducibility and resistance to the effects of gene loss or rearrangements (11, 12). cgSNP approaches, based on variant calling against a reference genome, can provide even higher resolution while allowing the exclusion of recombinant regions (12).

Here, we present the first ad hoc cgMLST scheme for *C. butyricum*, developed using the open-source chewBBACA pipeline (13) and a data set comprising 200 newly sequenced strains and 97 publicly available genomes. We applied cgMLST and cgSNP analyses to investigate strain-level phylogeny and epidemiology, including differentiation of isolates from NEC cases and controls across multiple geographic and temporal settings.

## MATERIALS AND METHODS

### Study and sample collection

Stool samples were collected from preterm neonates (<37 weeks’ gestation) enrolled in multiple French clinical trials conducted between 2008 and 2023, across 13 neonatal intensive care units (NICUs) (14, 15). Additional samples were collected from NICUs in southeastern France between 2009 and 2023 (16–18). In case–control cohorts, NEC cases and matched controls were selected within the same NICU, matched on gestational age, birth weight, feeding type, and mode of delivery. In outbreak investigations, asymptomatic carriers were matched to NEC cases based additionally on sex, day of life, prior antibiotic exposure, and NICU admission. NEC diagnosis followed modified Bell’s criteria (stages I–III), with confirmed cases defined as stages II and III based on clinical, radiological, surgical, or autopsy findings (19). Stool samples were collected from diapers as previously described and stored at –80°C (16, 20).

### Bacterial isolation and identification

All new clinical isolates included in this study were obtained from the strain collections of the Laboratoire de Microbiologie, U1139 (FRPM), Faculté de Pharmacie de Paris (Université Paris Cité, France), and the IHU Méditerranée Infection (Université de Marseille, France). Isolation procedures followed previously established protocols (16, 20). Briefly, frozen stool samples stored were homogenized, serially diluted, and plated onto selective agar media. Plates were incubated anaerobically (CO_2_:H_2_:N_2_, 10:10:80) using an anaerobic chamber (Don Whitley Scientific, UK). Colonies were identified by matrix-assisted laser desorption/ionization time-of-flight mass spectrometry (Bruker Daltonics S.A.). Isolates were stored in brain heart infusion broth supplemented with 20% (vol/vol) glycerol at –80°C. For culture, bacterial liquid cultures were grown in TGYH broth (tryptone 30 g/L, glucose 5 g/L, yeast extract 20 g/L, and hemin 5 mg/L) for 48 hours at 37°C under anaerobic conditions.

### Genomic DNA extraction and whole-genome sequencing

For the sequencing of the 200 newly isolated *C. butyricum* strains included in this study, genomic DNA was extracted from 24 h bacterial liquid cultures using the DNeasy UltraClean Microbial Kit (Qiagen, Courtaboeuf, France) according to the manufacturer’s instructions. Whole-genome sequencing of these 200 *C*. *butyricum* strains was performed at the Biomics Platform (Institut Pasteur, Paris, France) and the IHU Méditerranée Infection. DNA libraries were prepared using the Nextera XT DNA Library Prep Kit (Illumina, San Diego, CA, USA) and sequenced on Illumina HiSeq or NextSeq 500 platforms (2 × 150 bp paired end). For the reference strain *C. butyricum* DSMZ 10,702^T^/ATCC 19398, whole-genome sequencing was performed using both Illumina short reads and Oxford Nanopore Technologies long reads (21). Illumina reads were processed using Fastp v.0.23.4, with quality filtering parameters set to remove reads containing >5 ambiguous bases, low-quality bases (*Q* ≤ 30), and adapter contamination. Filtered reads were then assembled *de novo* using Unicycler v.0.4.8. For the reference genome, a Unicycler default hybrid assembly was performed.

### Genome assembly quality

Assembly quality was assessed using QUAST v.5.3.0 (reporting number of contigs, N50, largest contig length, total size, GC%). Genome completeness and contamination were estimated with CheckM v.1.2.2. In the present study, only “high-quality draft” genomes were included for analysis (≥90% completeness, ≤5% contamination, 5S/16S/23S rRNAs present, and ≥18 tRNAs) (22). In addition, we excluded publicly available genomes with stretches of unresolved bases (Ns). Genomic similarity was measured using the pairwise average nucleotide identity (ANI) computed with FastANI v.1.34 (23) to generate a symmetric ANI matrix. The matrix was visualized as a clustered heatmap in R v.4.3.2 (pheatmap v.1.0.13) (24).

### The cgMLST scheme construction

The *C. butyricum* cgMLST scheme was generated using chewBBACA v.3.9.9 (25) with 297 high-quality draft genomes (200 sequenced in this study, 97 public). The coding DNA sequence of the 297 genomes was predicted using Prodigal v.2.6.3, and pairwise all-against-all BLASTP (BLAST+ v.2.13.0) comparisons were performed to cluster loci using a BLAST score ratio threshold of 0.6. Loci were grouped into allelic profiles, and paralogous loci were identified with chewBBACA’s AlleleCall and RemoveGenes modules then excluded. The resulting whole-genome multilocus sequence typing scheme contained 9,711 loci, from which 39 paralogs were removed.

Core genes were defined as loci present in ≥95% of genomes, as implemented in chewBBACA’s ExtractCgMLST module with default parameters, producing the cgMLST-95 scheme. This filtering resulted in a final cgMLST scheme of 2,621 core genome loci and 7,090 accessory genome loci. The complete schema file is available at cgMLST.org, and targets are also listed in Table S1.

To explore genetic relationships among isolates, allelic profiles for cgMLST-95 loci were used to compute genetic distances. Neighbor-joining (StandardNJ) trees were built in GrapeTree v.1.5.0 (26) from the allelic distance matrix.

Minimum spanning trees (MSTs) were constructed in GrapeTree and in PHYLOViZ v.2.0 (27) using the goeBURST nLV algorithm. All phylogenies were visualized in iTOL v.6 (28).

### cgSNP analysis

cgSNP analysis was performed using raw reads from the 297 *C*. *butyricum* genomes. The complete genome of *C. butyricum* DSMZ10702^T^ was used for read alignment and variant calling. Single-nucleotide polymorphism (SNP) calling was performed with Snippy v.4.6.0 (https://github.com/tseemann/snippy), with default parameters.

The resulting core SNP alignment was processed with Gubbins v.3.0.0 (five iterations, default parameters) to mask regions of elevated SNP density indicative of recombination (29). The recombination-filtered core alignment was used to infer a maximum likelihood phylogeny with RAxML-NG v.1.1.0 under the GTR + G model and 1,000 non-parametric bootstrap replicates. Trees were visualized and annotated in iTOL v.6. Phylogenetic reconstruction was performed using RAxML-NG, applying the GTR-GAMMA model and 1,000 bootstrap replicates to generate a maximum likelihood tree. The resulting phylogenetic tree was visualized and annotated using iTOL v.6 (28). Pairwise SNP distances between isolates were calculated from the Gubbins-filtered alignment using Snp-dists v.0.8.2 (output in SNP counts) (https://github.com/tseemann/snp-dists). The resulting distance matrix was clustered using hierarchical clustering in R v.4.3.2 to produce a distance-based dendrogram.

### Statistical analysis

XLSTAT v.2014.5.03 was used for statistical analysis. Fisher’s exact test or Pearson’s chi-squared test was used to determine non-random associations between variables, with significance set at *P* < 0.05.

## RESULTS

### *C. butyricum* population characteristics

We analyzed 297 *C*. *butyricum* genomes, including 200 newly sequenced isolates and 97 publicly available genomes (Table S1). Over half of the newly sequenced isolates (*n* = 101, 51%) originated from neonates with NEC. The publicly available genomes represented diverse sources: plants (*n* = 53, 18.0%), adult humans (*n* = 11, 4.0%), unidentified origin (*n* = 11, 4.0%), animals (*n* = 10, 3.0%), environmental samples (*n* = 4, 1.0%), human infants (*n* = 8, 3.0%), and probiotic (*n* = 2, 0.7%).

### Whole-genome data

Genome sizes ranged from 4.1 to 5.8 Mb (mean ± SD: 4.6 ± 0.22 Mb), with a mean G + C content of 28.65% ± 0.11%. Predicted protein-coding genes numbered 3,613–5,584 (mean ± SD: 4,169 ± 284). Pairwise whole-genome comparisons revealed ANI values spanning 97.2% (most divergent) to 100% (most similar) (Fig. S1).

### Development and evaluation of the *C. butyricum* cgMLST scheme

**Fig 1:**
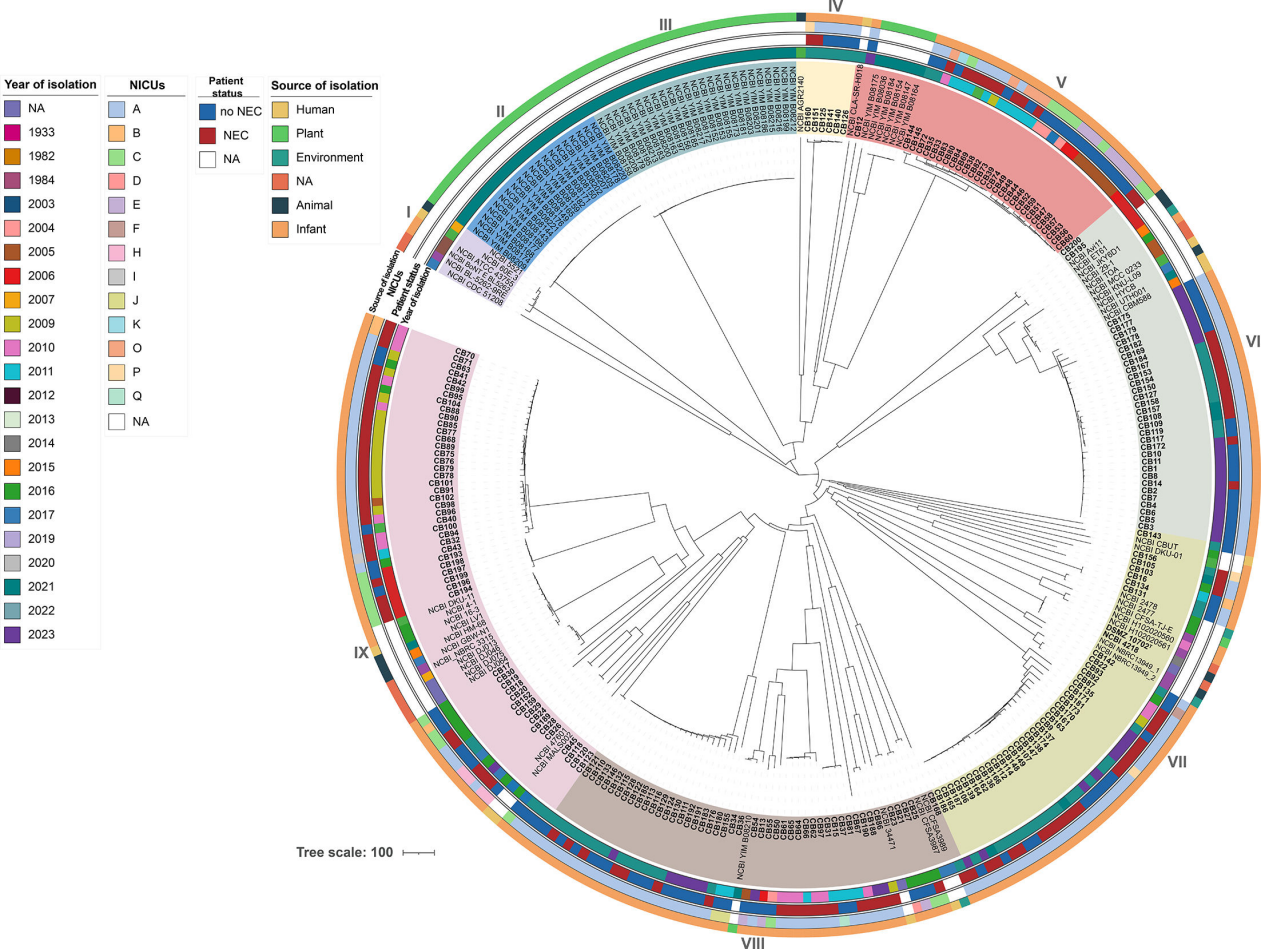
Neighbor-joining cgMLST phylogeny of the 297 *C*. *butyricum* strains. Year of isolation, NICUs, NEC status of the patient (no NEC or NEC), and source of isolation of the samples are given for each strain. The *C. butyricum* type strain DSMZ10702^T^ is the reference strain (bold). Newly sequenced strains from preterm infants are shown in bold. Roman numerals indicate the number assigned to the clade. NA, data not available. NCBI_BoNT E BL5262 and NCBI BL-5262-9RE genome sequences are from the same strain.

From 297 *C*. *butyricum* genomes, we developed a cgMLST scheme comprising 2,621 genes (Table S2). Neighbor-joining phylogeny based on cgMLST data resolved nine clades (Fig. 1): I (*n* = 6, 2%), II (*n* = 20, 7%), III (*n* = 25, 8%), IV (*n* = 7, 2%), V (*n* = 35, 12%), VI (*n* = 42, 14%), VII (*n* = 49, 16%), VIII (*n* = 50, 17%), and IX (*n* = 63, 21%).

The reference strain DSMZ 10702^T^ was assigned to clade VII. Clades II, III, and IV showed temporal clustering, while clades VIII and IX displayed the greatest temporal diversity. Plant-derived strains were concentrated in clades II and III (88% of all plant isolates), with few in clades V and VIII. Clinical isolates from NICU A were significantly overrepresented (*P* < 0.001), particularly in clades VI (*P* = 0.01), VII (*P* < 0.001), and VIII (*P* = 0.01). The two probiotic strains, NCBI CBM588 and NCBI TOA, were assigned to clade VI.

All five botulinum neurotoxin type E (BoNT/E)-producing strains formed clade I, showing high allelic similarity and clear separation from other strains, consistent with clonal expansion or recent common ancestry (Fig. 1; Fig. S2). MST trees based on core genome allelic profiles and the distribution of strains according to the country of origin, year of isolation, and source of isolation are provided in Fig. S3, S4 and S5, respectively. MST analysis revealed significant overrepresentation of strains from France and China (*P* < 0.001 for both), as well as isolates from preterm infants and plants (*P* < 0.001). Preterm strains from France were found within clades that also included strains from other human, animal, and plant sources, with no clear segregation by year or geographic origin. Strains from plant roots in China in 2021 formed a geographically and temporally restricted cluster. BoNT/E strains were split into two genetically distinct human-derived clusters. Network analysis using the goeBURST nLV algorithm showed that of the 53 plant strains, 7 and 10 had the same cgMLST profile as NCBI YIM B08212 and NCBI YIM B08178, respectively. Other strains had the same cgMLST profile, such as BoNT/E NCBI_60E3 and NCBI_5521, CB154 and CB150, and CB5 and CB3.

### cgSNP analysis and comparison with cgMLST

**Fig 2:**
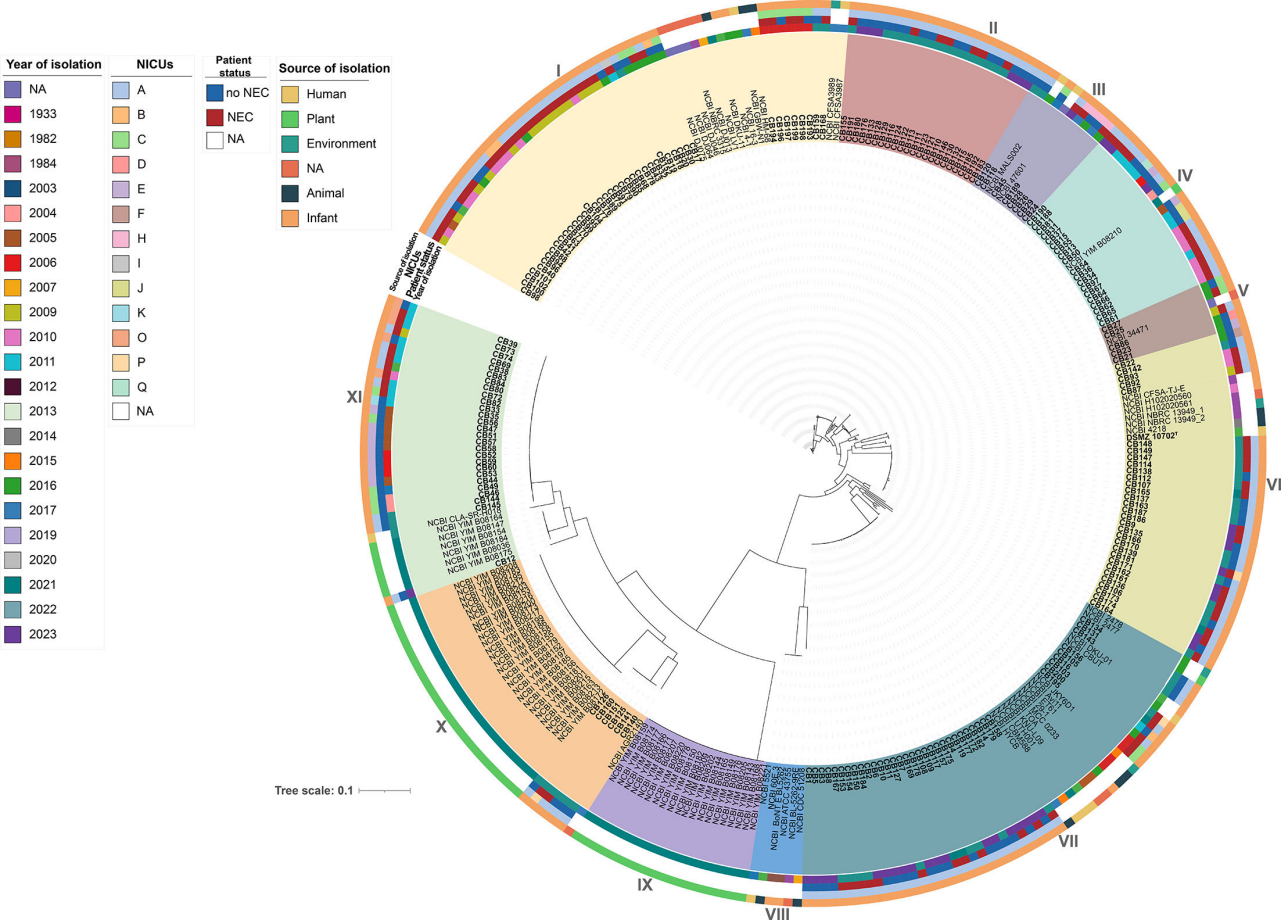
Maximum likelihood cgSNP tree of the 297 *C*. *butyricum* strains. Year of isolation, NICU, NEC status of the patient (no NEC or NEC) and source of isolation of the samples are given for each strain. The *C. butyricum* type strain DSMZ10702^T^ is the reference strain (bold). Newly sequenced strains from preterm infants are shown in bold. Roman numerals indicate the number assigned to the clade. NA, data not available. NCBI_BoNT E BL5262 and NCBI BL-5262-9RE genome sequences are from the same strain.

After removal of predicted recombinant regions, cgSNP analysis identified 87,681 SNPs. The maximum likelihood phylogeny resolved 11 clusters (Fig. 2): I (*n* = 55, 19%), II (*n* = 22, 7%), III (*n* = 10, 3%), IV (*n* = 20, 7%), V (*n* = 6, 2%), VI (*n* = 38, 13%), VII (*n* = 53, 18%), VIII (*n* = 6, 2%), IX (*n* = 20, 7%), X (*n* = 32, 11%), and XI (*n* = 35, 12%).

The reference strain DSMZ 10702^T^ was assigned to cluster VI. Both probiotic strains NCBI CBM588 and NCBI TOA were assigned to cluster VII. BoNT/E-producing strains clustered identically in both cgSNP and cgMLST analyses. For the remaining isolates, cgSNP revealed two additional clusters (III and V) compared with cgMLST, reflecting its higher resolution. Clinical isolates from cgMLST clade IV were assigned to cgSNP cluster X containing plant-derived strains (Fig. 1 and 2). While overall strain distributions were similar, tree topologies differed, particularly for cgSNP clusters II, V, and X. Pairwise SNP distance analysis (Fig. S6) showed some isolates differed by <20 SNPs, consistent with recent transmission or a shared source, whereas others differed by >500 SNPs, indicating long-term divergence.

### cgMLST- and cgSNP-based analyses of *C. butyricum* strains isolated from patients with or without NEC

Clinical isolates were significantly more frequent in NICU A (*n* = 145) and NICU C (*n* = 21) compared with other NICUs (*P* < 0.001). In the cgMLST analysis, clade XI contained significantly more NEC-associated strains than clades V (*P* = 0.01) and VI (*P* = 0.04). In the cgSNP analysis, NEC strains were significantly enriched in cluster I compared with clusters II (*P* = 0.002), IV (*P* = 0.003), VII (*P* = 0.02), and IX (*P* = 0.001), and in cluster VI compared with cluster XI (*P* = 0.03).

When NEC and non-NEC isolates were compared overall, cgMLST clade distribution showed no significant association with NEC status (*P* = 0.068), whereas cgSNP clustering was significantly associated with NEC (*P* = 0.016). Within certain clades, genomes formed tightly grouped, near-clonal lineages (Fig. 1 and 2).

**Fig 3:**
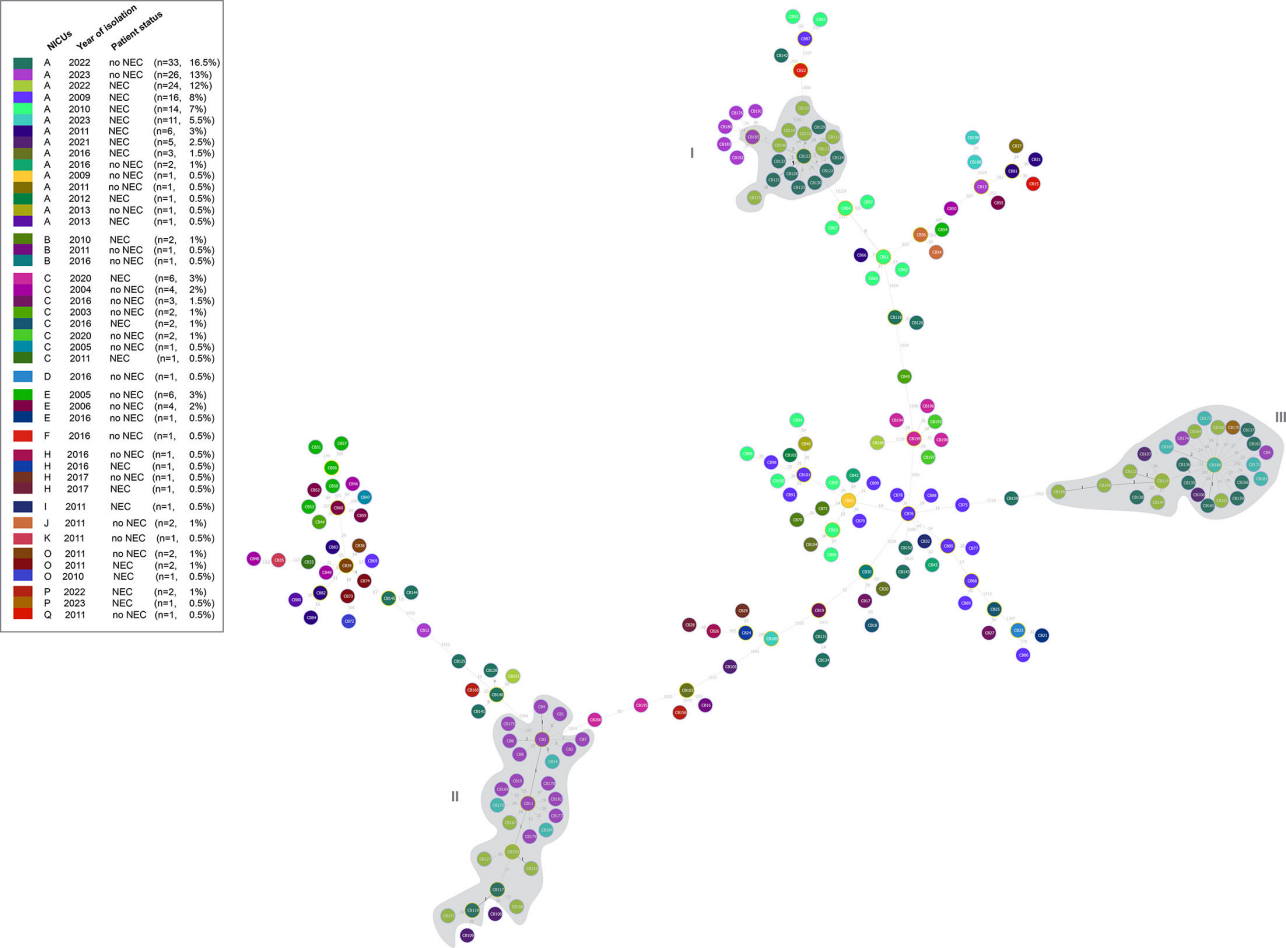
Minimum-spanning tree generated with the goeBURST nLV algorithm for the cgMLST-95 profiles of 200 *C*. *butyricum* clinical isolates. Colors were attributed to nodes according to patient status (NEC or no NEC), year of isolation, and NICU. Nodes highlighted in yellow represent clonal complexes. Gray color highlight grouped strains linked with a majority of strains with **≤** 25 differences between profiles.

The goeBURST nLV minimum spanning tree (Fig. 3), generated from cgMLST-95 profiles, suggested possible intra-NICU clonal spread. In NICU A, isolates collected in 2022–2023—spanning both NEC and non-NEC cases—were grouped into the same clonal complexes (groups I and II). Notably, most NEC and non-NEC isolates from 2022 in group I differed by fewer than 25 alleles, indicating recent common ancestry.

## DISCUSSION

In this study, we analyzed 200 newly sequenced *C. butyricum* genomes, including a majority from preterm neonates with NEC, alongside environmental, plant, and animal isolates. To our knowledge, this constitutes the largest genomic data set for this species to date and enables the development of the first dedicated cgMLST scheme.

Genomic features were consistent with prior reports (3), with genome sizes ranging from 3.7 to 5.2 Mb and a mean GC content of 28.6%, demonstrating broad genomic stability despite variation in accessory genes. Population structure analysis revealed clearly defined genetic clusters, highlighting epidemiologically relevant sublineages. While both cgMLST and cgSNP identified major lineages, cgSNP provided superior resolution, uncovering NEC-associated clusters that cgMLST did not detect.

Notably, several clades contained near-clonal strains, often restricted to individual NICUs. This observation, together with previous reports of NICU-specific clones (17, 30) and documented NEC outbreaks (18), strongly supports the existence of local persistence and potential healthcare-associated transmission, likely facilitated by environmental reservoirs or patient transfers. Such findings demonstrate the critical value of high-resolution genomic surveillance in detecting clinically significant transmission events that would otherwise remain hidden.

Although our data set was largely limited to French NICUs, potentially introducing sampling bias, the integrated cgMLST–cgSNP approach proved robust for both broad lineage delineation and fine-scale clonality assessment. Routine implementation of these genomic tools in neonatal care could enable rapid outbreak detection, guide targeted infection control interventions, and ultimately reduce the risk of NEC linked to pathogenic *C. butyricum* strains in vulnerable preterm infants.

## Data Availability

All newly generated raw Illumina and Oxford Nanopore sequence reads, along with assembled genomes, have been deposited in the Sequence Read Archive Database at the National Center for Biotechnology Information under BioProject accession no. PRJEB90282. Individual accession numbers for each sample are provided in Table S1.

## SUPPLEMENTAL MATERIAL

Supplemental figures spectrum.02619-25-s0001.pdf Figures S1 to S6.

Supplemental tables spectrum.02619-25-s0002.pdf Tables S1 and S2.

ASM does not own the copyrights to Supplemental Material that may be linked to, or accessed through, an article. The authors have granted ASM a non-exclusive, world-wide license to publish the Supplemental Material files. Please contact the corresponding author directly for reuse.
