# Supplementary material for: Whole-genome sequencing-based typing methods for Clostridium butyricum strains from clinical, animal, plant, and environmental sources
Source: Microbiol Spectr. 2025 Dec 12;14(2):e02619-25. doi: 10.1128/spectrum.02619-25 (PMC12889113; doi:10.1128/spectrum.02619-25)
Supplement: Supplemental tables — Tables S1 and S2. [file spectrum.02619-25-s0002.pdf]

Table S1. cgMLST profiles of the 297 *C. butyricum* strains examined in the study.

Strain ID	Year of isolation	NEC*	Origin	Country	Code City	Sequencing technology	Contigs/ genome close level	Size genome (bp)	Contigs N50	Contigs L50	G+C (%)	Completeness (%)	Contamination (%)	CDS	tRNA	rRNA	Repeated Regions (%)	Accession Number.
CB1	2023	0	Premature stools	France	A	Illumina	3	4627543	3834491	1	28.78	98.27	0	4099	85	36	1	PRJEB90282
CB2	2023	0	Premature stools	France	A	Illumina	4	4630718	3834494	1	28.77	98.27	0	4102	85	36	1	PRJEB90282
CB3	2023	0	Premature stools	France	A	Illumina	3	4627559	3834502	1	28.78	98.27	0	4091	85	36	1	PRJEB90282
CB4	2023	0	Premature stools	France	A	Illumina	5	4627098	3804519	1	28.78	98.27	0	4094	86	36	1	PRJEB90282
CB5	2023	0	Premature stools	France	A	Illumina	3	4627556	3834498	1	28.78	98.27	0	4090	85	36	1	PRJEB90282
CB6	2023	0	Premature stools	France	A	Illumina	3	4627554	3834499	1	28.78	98.27	0	4092	85	36	1	PRJEB90282
CB7	2023	0	Premature stools	France	A	Illumina	3	4628665	3835610	1	28.78	98.27	0	4099	85	36	1	PRJEB90282
CB8	2023	0	Premature stools	France	A	Illumina	5	4632613	3832894	1	28.77	98.27	0	4110	85	36	1	PRJEB90282
CB9	2023	0	Premature stools	France	A	Illumina	11	5240598	3077623	1	28.74	98.27	0	4916	83	30		PRJEB90282
CB10	2023	0	Premature stools	France	A	Illumina	4	4629910	3832979	1	28.77	98.27	0	4103	85	36	1	PRJEB90282
CB11	2023	0	Premature stools	France	A	Illumina	3	4627597	3834539	1	28.78	98.27	0	4091	86	36	1	PRJEB90282
CB12	2023	0	Premature stools	France	A	Illumina	6	4606370	3801658	1	28.74	98.27	0	4018	72	33		PRJEB90282
CB13	2023	0	Premature stools	France	A	Illumina	3	4701415	3868116	1	28.76	98.27	0	4203	88	36		PRJEB90282
CB14	2023	1	Premature stools	France	A	Illumina	4	4662311	3829872	1	28.76	98.27	0	4152	87	33	1	PRJEB90282
CB20	2016	1	Premature stools	France	A	Illumina	123	4554357	92334	15	28.54	98.27	0	4130	81	10		PRJEB90282
CB31	2011	1	Premature stools	France	A	Illumina	66	4493886	182369	8	28.49	98.27	0	3988	58	10		PRJEB90282
CB37	2011	0	Premature stools	France	A	Illumina	96	4541146	182370	8	28.5	98.27	0	4060	82	9		PRJEB90282
CB40	2013	0	Premature stools	France	A	Illumina	96	4714400	154343	9	28.68	98.27	0	4245	88	35		PRJEB90282
CB41	2009	0	Premature stools	France	A	Illumina	71	4698980	216273	7	28.59	98.27	0	4237	77	15		PRJEB90282
CB42	2016	0	Premature stools	France	A	Illumina	59	4690796	208490	9	28.55	98.27	0	4239	76	11		PRJEB90282
CB43	2016	0	Premature stools	France	A	Illumina	52	4670938	195169	9	28.54	98.27	0	4234	64	11		PRJEB90282
CB61	2010	1	Premature stools	France	A	Illumina	81	4648064	169507	8	28.53	98.27	0	4223	80	11		PRJEB90282
CB62	2010	1	Premature stools	France	A	Illumina	81	4648403	169507	8	28.52	97.41	0	4230	77	11		PRJEB90282
CB63	2010	1	Premature stools	France	A	Illumina	76	4667517	152473	11	28.54	98.27	0	4222	84	9		PRJEB90282
CB64	2010	1	Premature stools	France	A	Illumina	83	4666341	155915	10	28.53	98.27	0	4269	77	8		PRJEB90282

CB65	2010	1	Premature stools	France	A	Illumina	168	4611641	49962	27	28.55	98.27	0	4189	73	9		PRJEB90282
CB66	2011	1	Premature stools	France	A	Illumina	163	4599670	50365	30	28.55	98.27	0	4177	68	8		PRJEB90282
CB67	2010	1	Premature stools	France	A	Illumina	26	4742905	364199	5	28.66	95.53	0	5289	74	16		PRJEB90282
CB68	2009	1	Premature stools	France	A	Illumina	97	4711741	101477	15	28.54	98.27	0	4283	82	9		PRJEB90282
CB69	2009	1	Premature stools	France	A	Illumina	51	4498679	255182	7	28.55	98.27	0	3964	84	11	1	PRJEB90282
CB75	2009	1	Premature stools	France	A	Illumina	47	4636623	214563	8	28.54	98.27	0	4179	87	13		PRJEB90282
CB76	2009	1	Premature stools	France	A	Illumina	58	4656895	228436	7	28.62	98.27	0	4176	85	27		PRJEB90282
CB77	2009	1	Premature stools	France	A	Illumina	76	4625276	159482	11	28.55	98.27	0	4175	87	9		PRJEB90282
CB78	2009	1	Premature stools	France	A	Illumina	113	4662337	116723	13	28.6	98.27	0	4199	83	17		PRJEB90282
CB79	2009	1	Premature stools	France	A	Illumina	116	4686265	122649	11	28.61	98.27	0	4229	79	18		PRJEB90282
CB80	2013	1	Premature stools	France	A	Illumina	49	4532832	420200	4	28.62	98.27	0	3975	87	15	1	PRJEB90282
CB81	2011	1	Premature stools	France	A	Illumina	48	4559174	213440	8	28.52	98.27	0	4081	80	12		PRJEB90282
CB82	2011	1	Premature stools	France	A	Illumina	106	4555369	262728	6	28.76	98.27	0	3998	99	22	1	PRJEB90282
CB83	2011	1	Premature stools	France	A	Illumina	97	4475575	132885	10	28.56	98.27	0	3923	84	13	1	PRJEB90282
CB84	2011	1	Premature stools	France	A	Illumina	33	4519546	517787	3	28.53	98.27	0	3984	84	10	1	PRJEB90282
CB85	2009	1	Premature stools	France	A	Illumina	58	4633142	209766	8	28.54	98.27	0	4174	84	13		PRJEB90282
CB86	2009	1	Premature stools	France	A	Illumina	84	4547659	143195	11	28.61	98.27	0	4101	85	19		PRJEB90282
CB87	2009	1	Premature stools	France	A	Illumina	71	4726805	266880	5	28.66	98.27	0	4202	97	24		PRJEB90282
CB88	2009	1	Premature stools	France	A	Illumina	100	4717585	201198	7	28.57	98.27	0.78	4258	88	11		PRJEB90282
CB89	2009	1	Premature stools	France	A	Illumina	62	4747213	226782	8	28.62	98.27	0	4304	84	16		PRJEB90282
CB90	2010	1	Premature stools	France	A	Illumina	59	4697440	208044	8	28.56	98.27	0	4258	80	11		PRJEB90282
CB91	2009	1	Premature stools	France	A	Illumina	74	4691387	152473	9	28.57	98.27	0	4249	86	14		PRJEB90282
CB92	2010	1	Premature stools	France	A	Illumina	55	4651126	184146	8	28.58	98.27	0	4157	84	14		PRJEB90282
CB93	2010	1	Premature stools	France	A	Illumina	252	5164841	75526	21	28.6	98.27	0	4656	87	11		PRJEB90282
CB94	2010	1	Premature stools	France	A	Illumina	164	4654240	91412	16	28.59	98.27	1.88	4185	80	11		PRJEB90282
CB95	2010	1	Premature stools	France	A	Illumina	71	4659172	160702	10	28.55	98.27	0	4209	80	13		PRJEB90282
CB96	2010	1	Premature stools	France	A	Illumina	94	4680205	113411	13	28.55	98.27	0	4236	83	10		PRJEB90282
CB97	2010	1	Premature stools	France	A	Illumina	245	4520161	41394	33	28.61	98.27	0	4135	72	10		PRJEB90282
CB98	2009	1	Premature stools	France	A	Illumina	82	4718311	198390	8	28.67	98.27	0	4238	84	35		PRJEB90282

CB99	2009	1	Premature stools	France	A	Illumina	91	4676122	140698	12	28.55	98.27	0	4227	78	11		PRJEB90282
CB100	2010	1	Premature stools	France	A	Illumina	85	4677749	130666	12	28.55	98.27	0	4235	80	11		PRJEB90282
CB101	2009	1	Premature stools	France	A	Illumina	64	4694866	218993	6	28.58	98.27	0	4238	92	14		PRJEB90282
CB102	2012	1	Premature stools	France	A	Illumina	154	4629832	81178	18	28.57	98.27	0	4164	78	10		PRJEB90282
CB103	2016	1	Premature stools	France	A	Illumina	100	4591026	97383	17	28.6	98.27	1.72	4121	80	12		PRJEB90282
CB104	2016	1	Premature stools	France	A	Illumina	81	4685615	153544	9	28.55	98.27	0	4244	81	11		PRJEB90282
CB105	2021	1	Premature stools	France	A	Illumina	2	4511530	3809540	1	28.74	98.27	0	3929	88	33		PRJEB90282
CB106	2021	1	Premature stools	France	A	Illumina	225	5051442	56958	27	28.6	98.27	0	4706	71	9		PRJEB90282
CB107	2021	1	Premature stools	France	A	Illumina	245	5040767	41635	36	28.6	98.27	0	4687	77	10		PRJEB90282
CB108	2021	1	Premature stools	France	A	Illumina	211	4675645	51093	24	28.69	98.27	0	4196	81	9	1	PRJEB90282
CB109	2021	1	Premature stools	France	A	Illumina	13	4682767	1529919	2	28.75	98.27	0	4225	86	33	1	PRJEB90282
CB110	2022	1	Premature stools	France	A	Illumina	90	4456562	83451	14	28.51	98.27	0	3974	77	12		PRJEB90282
CB111	2022	1	Premature stools	France	A	Illumina	2	4546817	3813736	1	28.74	98.27	0	4015	88	36		PRJEB90282
CB112	2022	1	Premature stools	France	A	Illumina	18	5217501	2729780	1	28.71	98.27	0	4901	73	23		PRJEB90282
CB113	2022	1	Premature stools	France	A	Illumina	2	4549775	3816667	1	28.74	98.27	0	4016	85	36		PRJEB90282
CB114	2022	1	Premature stools	France	A	Illumina	11	5223525	2730200	1	28.71	98.27	0	4909	85	24		PRJEB90282
CB115	2022	1	Premature stools	France	A	Illumina	76	4460641	108474	9	28.5	98.27	0	3974	72	12		PRJEB90282
CB116	2022	1	Premature stools	France	A	Illumina	59	4424945	225939	7	28.52	98.27	0	3920	78	12		PRJEB90282
CB117	2022	0	Premature stools	France	A	Illumina	3	4717524	3875696	1	28.77	98.27	0	4233	87	33	1	PRJEB90282
CB118	2022	0	Premature stools	France	A	Illumina	78	4649092	134880	10	28.53	98.27	0	4187	68	12		PRJEB90282
CB119	2022	0	Premature stools	France	A	Illumina	10	4682981	1384775	2	28.76	98.27	0	4183	87	33	1	PRJEB90282
CB120	2022	0	Premature stools	France	A	Illumina	185	4621990	47432	30	28.57	98.27	0	4157	66	12		PRJEB90282
CB121	2022	0	Premature stools	France	A	Illumina	58	4456678	188566	8	28.5	98.27	0	3975	69	11		PRJEB90282
CB122	2022	0	Premature stools	France	A	Illumina	55	4458438	225939	7	28.5	98.27	0	3977	77	10		PRJEB90282
CB123	2022	0	Premature stools	France	A	Illumina	57	4459651	225939	7	28.5	98.27	0	3977	78	11		PRJEB90282
CB124	2022	0	Premature stools	France	A	Illumina	56	4458620	225939	7	28.5	98.27	0	3978	67	11		PRJEB90282
CB125	2022	0	Premature stools	France	A	Illumina	88	4410491	91325	13	28.54	98.27	0	3965	83	9		PRJEB90282
CB126	2022	0	Premature stools	France	A	Illumina	2	4478030	3741774	1	28.77	98.27	0	3982	88	36		PRJEB90282
CB127	2022	1	Premature stools	France	A	Illumina	15	4674757	1605978	2	28.74	98.27	0	4181	86	30	1	PRJEB90282

CB128	2022	0	Premature stools	France	A	Illumina	7	4517026	3436137	1	28.69	98.27	0	4004	87	26		PRJEB90282
CB129	2022	0	Premature stools	France	A	Illumina	5	4549569	3766893	1	28.74	98.27	0	4021	85	36		PRJEB90282
CB130	2022	0	Premature stools	France	A	Illumina	11	4544201	1104463	2	28.72	98.27	0	4027	88	33		PRJEB90282
CB131	2022	0	Premature stools	France	A	Illumina	50	4503114	230941	7	28.52	98.27	0	3982	75	10		PRJEB90282
CB132	2022	0	Premature stools	France	A	Illumina	83	4456384	103461	13	28.5	98.27	0	3970	71	12		PRJEB90282
CB133	2022	0	Premature stools	France	A	Illumina	8	4512434	1261086	2	28.68	98.27	0	3995	88	29		PRJEB90282
CB134	2022	0	Premature stools	France	A	Illumina	69	4495385	150158	8	28.55	97.41	0	3972	85	12	1	PRJEB90282
CB135	2022	0	Premature stools	France	A	Illumina	30	5200897	538110	5	28.76	98.27	1.72	5115	87	27		PRJEB90282
CB136	2022	0	Premature stools	France	A	Illumina	19	5286370	1046963	2	28.74	98.27	0	4962	86	33		PRJEB90282
CB137	2022	0	Premature stools	France	A	Illumina	25	5198564	1405683	2	28.71	98.27	0	4861	85	23		PRJEB90282
CB138	2022	0	Premature stools	France	A	Illumina	207	5073858	57509	24	28.57	98.27	0	4729	75	11		PRJEB90282
CB139	2022	0	Premature stools	France	A	Illumina	241	5041444	47183	35	28.59	98.27	0	4701	77	10		PRJEB90282
CB140	2022	0	Premature stools	France	A	Illumina	25	4417376	384062	4	28.53	98.27	0	3971	82	10		PRJEB90282
CB141	2022	0	Premature stools	France	A	Illumina	33	4417559	260617	5	28.53	98.27	0	3969	75	10		PRJEB90282
CB142	2022	0	Premature stools	France	A	Illumina	76	4541588	144430	11	28.58	98.27	0	4063	75	11		PRJEB90282
CB143	2022	0	Premature stools	France	A	Illumina	62	4535386	195087	8	28.48	98.27	0	4008	78	10	1	PRJEB90282
CB144	2022	0	Premature stools	France	A	Illumina	90	4691920	103309	15	28.55	98.27	0	4223	84	10	1	PRJEB90282
CB145	2022	0	Premature stools	France	A	Illumina	4	4703826	3738318	1	28.7	98.27	0	4180	88	27	1	PRJEB90282
CB146	2022	1	Premature stools	France	A	Illumina	69	4457502	119517	10	28.5	98.27	0	3976	78	13		PRJEB90282
CB147	2022	1	Premature stools	France	A	Illumina	4	5242942	4194044	1	28.76	98.27	0	4946	83	30		PRJEB90282
CB148	2022	1	Premature stools	France	A	Illumina	3	5254546	4208128	1	28.76	98.27	0	4938	86	30		PRJEB90282
CB149	2022	1	Premature stools	France	A	Illumina	3	5254542	4208124	1	28.76	98.27	0	4937	86	30		PRJEB90282
CB150	2022	1	Premature stools	France	A	Illumina	3	4627828	3834706	1	28.78	98.27	0	4089	86	36	1	PRJEB90282
CB151	2022	1	Premature stools	France	A	Illumina	7	4400110	1720767	2	28.76	98.27	0	3921	87	33		PRJEB90282
CB152	2022	0	Premature stools	France	A	Illumina	2	4566867	3786389	1	28.74	98.27	0	4032	88	30		PRJEB90282
CB153	2022	1	Premature stools	France	A	Illumina	3	4627831	3834774	1	28.78	98.27	0	4091	86	36	1	PRJEB90282
CB154	2022	1	Premature stools	France	A	Illumina	3	4627763	3834705	1	28.78	98.27	0	4089	86	36	1	PRJEB90282
CB155	2022	1	Premature stools	France	A	Illumina	2	4568145	3755018	1	28.76	98.27	0	4012	88	36	1	PRJEB90282
CB157	2022	1	Premature stools	France	A	Illumina	11	4740717	2903715	1	28.89	98.27	0	4249	67	36	1	PRJEB90282

CB158	2022	1	Premature stools	France	A	Illumina	5	4807021	3732739	1	28.9	98.27	0	4335	83	36	1	PRJEB90282
CB159	2022	0	Premature stools	France	A	Illumina	76	5891383	326016	6	28.64	96.55	3.44	5584	88	12		PRJEB90282
CB161	2022	1	Premature stools	France	A	Illumina	43	5146269	348680	4	28.71	98.27	0	4822	85	24		PRJEB90282
CB162	2022	1	Premature stools	France	A	Illumina	2	5221185	5083973	1	28.75	98.27	0	4898	86	30		PRJEB90282
CB163	2022	0	Premature stools	France	A	Illumina	12	5206141	3385184	1	28.76	98.27	0	4906	84	30		PRJEB90282
CB164	2022	1	Premature stools	France	A	Illumina	3	5216158	4173289	1	28.75	98.27	0	4897	86	30		PRJEB90282
CB165	2022	0	Premature stools	France	A	Illumina	3	5225892	4179485	1	28.75	98.27	0	4903	86	30		PRJEB90282
CB166	2022	0	Premature stools	France	A	Illumina	10	5219754	1406288	2	28.75	98.27	0	4888	86	30		PRJEB90282
CB167	2022	1	Premature stools	France	A	Illumina	3	4627554	3834694	1	28.78	98.27	0	4103	86	36	1	PRJEB90282
CB168	2022	1	Premature stools	France	A	Illumina	7	4753756	2805317	1	28.74	98.27	0	4324	75	30		PRJEB90282
CB169	2023	0	Premature stools	France	A	Illumina	4	4670873	3874501	1	28.78	98.27	0	4175	85	36	1	PRJEB90282
CB171	2023	1	Premature stools	France	A	Illumina	28	5207638	3817789	1	28.78	98.27	1.72	5065	73	30		PRJEB90282
CB172	2023	1	Premature stools	France	A	Illumina	3	4625590	3832324	1	28.77	98.27	0	4104	66	36	1	PRJEB90282
CB173	2023	1	Premature stools	France	A	Illumina	10	5183187	3097446	1	28.75	98.27	0	4914	70	30		PRJEB90282
CB174	2023	0	Premature stools	France	A	Illumina	11	5239378	1414259	2	28.75	98.27	0	4935	78	30		PRJEB90282
CB175	2023	0	Premature stools	France	A	Illumina	34	4956926	1053148	2	28.8	98.27	0	4501	79	40	1	PRJEB90282
CB176	2023	0	Premature stools	France	A	Illumina	3	4609297	4587426	1	28.75	98.27	0	4128	70	36		PRJEB90282
CB177	2023	0	Premature stools	France	A	Illumina	4	4700958	3904462	1	28.79	98.27	0	4227	72	36	1	PRJEB90282
CB178	2023	0	Premature stools	France	A	Illumina	8	4665785	1050232	2	28.78	98.27	0	4179	79	36	2	PRJEB90282
CB179	2023	0	Premature stools	France	A	Illumina	8	4659214	962641	3	28.78	98.27	0	4176	66	36	1	PRJEB90282
CB180	2023	0	Premature stools	France	A	Illumina	13	4611041	1416513	2	28.76	98.27	0	4151	73	36		PRJEB90282
CB181	2023	1	Premature stools	France	A	Illumina	17	5215017	1473802	2	28.74	98.27	0	4931	66	30		PRJEB90282
CB182	2023	0	Premature stools	France	A	Illumina	3	4621654	3828596	1	28.75	98.27	0	4097	73	33	1	PRJEB90282
CB183	2023	0	Premature stools	France	A	Illumina	2	4603908	3812625	1	28.74	98.27	0	4118	70	36		PRJEB90282
CB184	2023	1	Premature stools	France	A	Illumina	3	4624452	3831394	1	28.76	98.27	0	4099	86	33	1	PRJEB90282
CB185	2023	0	Premature stools	France	A	Illumina	2	4606583	3814768	1	28.75	98.27	0	4106	88	36		PRJEB90282
CB186	2023	1	Premature stools	France	A	Illumina	4	5254543	4167883	1	28.76	98.27	0	4937	86	30		PRJEB90282
CB187	2023	1	Premature stools	France	A	Illumina	3	5254524	4208124	1	28.76	98.27	0	4939	86	30		PRJEB90282
CB188	2023	1	Premature stools	France	A	Illumina	5	4683030	3753412	1	28.8	98.27	0	4142	82	36		PRJEB90282

CB189	2023	1	Premature stools	France	A	Illumina	4	4646514	3830728	1	28.77	98.27	0	4064	88	36		PRJEB90282
CB190	2023	1	Premature stools	France	A	Illumina	2	4684370	3886876	1	28.8	98.27	0	4160	82	36		PRJEB90282
CB191	2023	0	Premature stools	France	A	Illumina	5	4637590	2455225	1	28.75	98.27	0	4174	66	36		PRJEB90282
CB192	2023	0	Premature stools	France	A	Illumina	4	4611668	1625149	2	28.74	98.27	0	4127	66	36		PRJEB90282
CB16	2011	0	Premature stools	France	B	Illumina	101	4576881	138057	12	28.55	98.27	0	4070	65	6		PRJEB90282
CB30	2016	0	Premature stools	France	B	Illumina	100	4550284	134799	13	28.55	98.27	0	4124	73	9		PRJEB90282
CB70	2010	1	Premature stools	France	B	Illumina	62	4674681	152473	10	28.53	98.27	0	4228	84	9		PRJEB90282
CB71	2010	1	Premature stools	France	B	Illumina	77	4690695	152473	11	28.54	98.27	0	4247	77	11		PRJEB90282
CB17	2016	0	Premature stools	France	C	Illumina	106	4524808	83987	14	28.55	98.27	0	4105	76	10		PRJEB90282
CB18	2016	1	Premature stools	France	C	Illumina	140	4517987	82846	14	28.54	98.27	0	4080	76	9		PRJEB90282
CB19	2016	0	Premature stools	France	C	Illumina	117	4523173	88174	14	28.57	98.27	0	4097	76	10		PRJEB90282
CB25	2016	1	Premature stools	France	C	Illumina	85	4472970	171570	9	28.53	98.27	0	4003	93	11		PRJEB90282
CB27	2016	0	Premature stools	France	C	Illumina	120	4426967	82056	13	28.56	98.27	0	3938	73	10		PRJEB90282
CB33	2011	1	Premature stools	France	C	Illumina	49	4478950	261162	4	28.49	98.27	0	3934	78	6	1	PRJEB90282
CB44	2003	0	Premature stools	France	C	Illumina	225	4342994	36041	28	28.63	98.27	0	3807	67	8		PRJEB90282
CB45	2003	0	Premature stools	France	C	Illumina	197	4512963	54708	26	28.61	98.27	0	4046	75	9		PRJEB90282
CB46	2004	0	Premature stools	France	C	Illumina	176	4423569	57125	20	28.6	98.27	0	3913	75	7	1	PRJEB90282
CB47	2005	0	Premature stools	France	C	Illumina	140	4360350	83258	15	28.57	96.55	0	3867	74	8	1	PRJEB90282
CB48	2004	0	Premature stools	France	C	Illumina	189	4107446	38745	26	28.74	98.27	0	3613	75	7	1	PRJEB90282
CB49	2004	0	Premature stools	France	C	Illumina	138	4399657	70830	18	28.61	98.27	0	3910	74	8	1	PRJEB90282
CB50	2004	0	Premature stools	France	C	Illumina	208	4532794	39613	32	28.55	98.27	0	4077	59	8		PRJEB90282
CB193	2020	0	Premature stools	France	C	Illumina	146	4620210	95334	15	28.53	98.27	0	4168	76	11		PRJEB90282
CB194	2020	1	Premature stools	France	C	Illumina	109	4635848	141128	11	28.53	98.27	0	4178	80	11		PRJEB90282
CB195	2020	1	Premature stools	France	C	Illumina	123	4477889	75524	20	28.59	98.27	0	3930	75	14		PRJEB90282
CB196	2020	1	Premature stools	France	C	Illumina	121	4622393	95335	15	28.54	98.27	0	4163	69	11		PRJEB90282
CB197	2020	0	Premature stools	France	C	Illumina	136	4555439	71956	22	28.55	96.55	0	4096	80	11		PRJEB90282
CB198	2020	1	Premature stools	France	C	Illumina	188	4527242	58113	25	28.63	98.27	0	4051	71	11		PRJEB90282
CB199	2020	1	Premature stools	France	C	Illumina	100	4633591	134056	10	28.53	98.27	0	4182	68	11		PRJEB90282
CB200	2020	1	Premature stools	France	C	Illumina	222	4538364	42246	29	29.23	98.27	2.87	3946	88	17		PRJEB90282

CB23	2016	0	Premature stools	France	D	Illumina	134	4806256	121759	11	28.63	98.27	0	4406	83	11		PRJEB90282
CB21	2016	0	Premature stools	France	E	Illumina	149	4801467	99506	14	28.63	98.27	0	4405	80	11		PRJEB90282
CB51	2005	0	Premature stools	France	E	Illumina	230	4245863	49131	25	28.72	96.55	0	3736	80	6	1	PRJEB90282
CB52	2006	0	Premature stools	France	E	Illumina	161	4456537	61467	22	28.57	98.27	0	3928	71	7	1	PRJEB90282
CB53	2005	0	Premature stools	France	E	Illumina	215	4289889	39829	36	28.68	98.27	0	3768	78	7	1	PRJEB90282
CB54	2005	0	Premature stools	France	E	Illumina	234	4485922	47928	26	28.59	96.55	0	4040	87	9		PRJEB90282
CB55	2006	0	Premature stools	France	E	Illumina	160	4629993	61277	23	28.57	98.27	0	4175	59	9		PRJEB90282
CB56	2005	0	Premature stools	France	E	Illumina	103	4380660	92119	16	28.59	98.27	0	3859	74	7	1	PRJEB90282
CB57	2005	0	Premature stools	France	E	Illumina	238	4375805	35360	29	28.66	98.27	0	3880	81	6	1	PRJEB90282
CB58	2005	0	Premature stools	France	E	Illumina	185	4369613	68602	21	28.65	98.27	0	3877	81	8		PRJEB90282
CB59	2006	0	Premature stools	France	E	Illumina	183	4419332	49130	26	28.58	90	0	3888	74	7	1	PRJEB90282
CB60	2006	0	Premature stools	France	E	Illumina	110	4480753	112247	13	28.51	98.27	0	3930	76	9	1	PRJEB90282
CB22	2016	0	Premature stools	France	F	Illumina	138	4573682	106465	14	28.56	98.27	0	4095	76	9		PRJEB90282
CB24	2016	1	Premature stools	France	H	Illumina	99	4573893	128240	13	28.55	98.27	0	4119	74	10		PRJEB90282
CB26	2016	0	Premature stools	France	H	Illumina	125	4684081	84595	15	28.58	98.27	0	4235	74	9		PRJEB90282
CB28	2017	1	Premature stools	France	H	Illumina	187	4667417	66363	23	28.59	98.27	0	4222	76	10		PRJEB90282
CB29	2017	0	Premature stools	France	H	Illumina	135	4545225	74873	19	28.59	98.27	0	4082	82	9		PRJEB90282
CB32	2011	1	Premature stools	France	I	Illumina	109	4647908	165507	10	28.53	98.27	0	4219	62	6		PRJEB90282
CB34	2011	0	Premature stools	France	J	Illumina	217	4497330	64216	22	28.63	98.27	0	4047	76	10		PRJEB90282
CB36	2011	0	Premature stools	France	J	Illumina	179	4558027	69390	18	28.63	98.27	0	4102	67	10		PRJEB90282
CB35	2011	0	Premature stools	France	K	Illumina	82	4204799	217992	8	28.63	98.27	0	3699	72	8	1	PRJEB90282
CB38	2011	0	Premature stools	France	O	Illumina	37	4501747	229798	6	28.51	98.27	0	3966	84	9	1	PRJEB90282
CB39	2011	0	Premature stools	France	O	Illumina	32	4513154	261732	4	28.52	98.27	0	3969	89	11	1	PRJEB90282
CB72	2010	1	Premature stools	France	O	Illumina	238	4513600	39875	30	28.57	98.27	0	3920	73	10	2	PRJEB90282
CB73	2011	1	Premature stools	France	O	Illumina	36	4501358	261732	5	28.51	98.27	0	3966	85	9	1	PRJEB90282
CB74	2011	1	Premature stools	France	O	Illumina	53	4504009	264062	3	28.51	98.27	0	3954	85	9	1	PRJEB90282
CB156	2022	1	Premature stools	France	P	Illumina	2	4749771	3932751	1	28.81	98.27	0	4232	88	33	1	PRJEB90282
CB160	2022	1	Premature stools	France	P	Illumina	3	4476098	3735607	1	28.75	98.27	0	4004	87	33		PRJEB90282
CB170	2023	1	Premature stools	France	P	Illumina	14	5243530	3136741	1	28.76	98.27	0	4959	86	30		PRJEB90282

CB15	2011	0	Premature stools	France	Q	Illumina	85	4456450	172922	9	28.52	98.27	0	3977	75	6		PRJEB90282
NCBI 60E.3	2013	NA	Stools	USA	R	Illumina	123	4540447	81014	17	28.81	90.36	0	3969	80	50		SAMN02596761
NCBI 2477	1982	NA	Cotton wood tree	USA	R	Illumina	3	4607921	3850094	1	28.77	94.82	0	4068	89	36	1	PRJEB90282
NCBI 2478	1982	NA	Lake sediment	USA	R	Illumina	3	4544883	3850094	1	28.77	94.82	0	4006	87	35	1	PRJEB90282
NCBI 4218	NA	NA	NA	NA	R	Illumina	4	4707145	3922832	1	28.74	98.27	0	4218	88	36		PRJEB90282
**DSMZ10702[T]**	2014	NA	**Reference strain** Intestine of pig	China	R	Illumina/ MinION	4	4650232	3865915	1	28.77	98.27	0	4179	88	36		PRJEB90282
NCBI 34471	NA	NA	Human tissue, tibia	NA	R	Illumina	2	4630841	3837749	1	28.88	98.27	0	4108	88	30		PRJEB90282
NCBI 47601	2003	NA	Human blood	Sweden	R	Illumina	4	4629848	3849433	1	28.77	98.27	0	4068	88	36		PRJEB90282
NCBI AGR2140	2013	NA	Rumen	USA	R	Illumina	42	4549947	237318	7	28.51	98.27	0	4063	56	8		SAMN02441161
NCBI CDC_51208	NA	NA	Infant with botulism	US	R	Illumina	3	4639914	3809831	1	28.69	98.27	0	4103	89	36	1	SAMN04262337
NCBI_5521	2007	NA	Infant with botulism	Italy	R	Illumina	123	4540447	81014	17	28.81	90.36	0	3969	80	50		SAMN02436238
DKU-01	2013	NA	Asymptomatic preterm infants stools	South-Korea	R	Illumina	79	4519722	108221	13	28.62	98.27	0	3989	60	17		SAMN02469601
NCBI BoNT E BL5262 = NCBI BL-5262-9RE	1984	NA	Infant with botulism	Italy	R	454 GS20	13	4622393	757	2	28.5	98.27	0	4226	85	39	1	SAMN02470281
NCBI HM-68	2015	NA	Healthy chicken intestine	Mongolia	R	Illumina	2	4604758	3835983	1	28.64	98.27	0	4145	76	21		SAMN03272540
NCBI KNU-L09	2013	NA	Fecal sample	South-Korea	R	Illumina	2	4627894	3824894	1	28.74	98.27	0	4098	88	33	1	SAMN04293668
NCBI JKY6D1	2015	NA	Pit mud of a Chinese liquor factory	China	R	Illumina; PacBio	3	4618327	3819894	3	28.73	97.93	0	4113	88	27	1	SAMN04285352
NCBI TOA	2012	NA	Probiotics	India	R	Illumina	3	4597202	3794139	1	28.67	98.27	0	4066	65	24	1	SAMN04527051
NCBI 29-1	2016	NA	First stool of the newborn	South-Korea	R	PacBio	2	4608805	3814519	1	28.73	98.27	0	4315	87	33	1	SAMN11489316
NCBI_4-1	2016	NA	First stool of the newborn	South-Korea	R	PacBio	2	4636588	3867296	1	28.76	98.27	0	4148	88	36		SAMN11489284
NCBI_NBRC 13949_1	2019	NA	Mock microbial communities	Japan	R	Illumina; ONT	4	4705096	3920778	1	28.74	98.27	0	4215	82	36		SAMD00169824
NCBI_NBRC 3315	2019	NA	NA	Japan	R	Illumina	62	4529872	168326	10	28.52	98.27	0	4098	58	7		SAMD00177824
NCBI NBRC 13949_2	2019	NA	Intestine of pig	Japan	R	Illumina	95	4590668	106338	12	28.5	98.27	0.31	4152	66	7		SAMD00177826
NCBI CFSA3987	2017	NA	Human stool	China	R	PacBio	2	4746849	3864433	1	28.81	98.27	0	4230	88	36		SAMN10285205
NCBI CFSA3989	2017	NA	Environment swab	China	R	PacBio	2	4746782	3864393	1	28.81	98.27	0	4217	88	36		SAMN10285378
NCBI H102020561	2019	NA	NA	England	R	Illumina; ONT	4	4651483	72479	1	28.48	98.27	0	4186	88	12		SAMN10868102
NCBI H102020560	2010	NA	Infant with botulism	England	R	Illumina	129	4654755	89937	16	28.48	98.27	0	4186	88	12		SAMN10822382
NCBI ATCC 43755	1984	NA	Infant with botulism	Italy	R	Illumina	134	4502098	81170	16	28.5	98.27	0	4027	82	14		SAMN11521329
NCBI BL-5262-9RE = NCBI BoNT E BL5262	2019	NA	Infant with botulism	Italy	R	Illumina	90	4667289	149957	11	28.47	98.27	0	4175	84	14	1	SAMN11521272
NCBI 16-3	2016	NA	Human stool	South-Korea	R	PacBio	2	4630815	3861515	1	28.74	98.27	0	4143	88	33		SAMN14846469

NCBI DJ064	NA	NA	NA	NA	R	PacBio		4640368	2842956	1	28.74	98.27	0	4154	88	33		SAMN05421551
NCBI HYCB	2017	NA	Gut chicken	China	R	Illumina	70	4517236	138316	10	28.53	98.27	0	4021	84	10	1	SAMN15338493
NCBI DJ046	NA	NA	NA	NA	R	PacBio	3	4657562	2941950	1	28.74	98.27	0	4187	88	33		SAMN05421549
NCBI DJ075	NA	NA	NA	NA	R	PacBio	2	4629054	3859765	1	28.74	98.27	0	4145	87	33		SAMN06297357
NCBI DJ013	2007	NA	NA	New-Zealand	R	Illumina	59	4528981	165212	11	28.5	98.27	0	4094	46	4		SAMN14588124
NCBI CBUT	2006	NA	Human stool	USA	R	Illumina	2	4488390	3782283	1	28.78	98.27	0	3945	88	36		SAMN18751771
NCBI CFSA-TJ-E	2019	NA	Stool from infant	China	R	Illumina	2	4697138	3949987	1	28.71	98.27	0	4197	88	33		SAMN14908714
NCBI LV1	2021	NA	Intestinal	China	R	Illumina	2	4625068	3855747	1	28.72	98.27	0	4155	88	30		SAMN32300840
NCBI MALS002	2016	NA	Human	India	R	Illumina	125	4549655	107911	13	28.65	98.27	0	4186	84	23		SAMN26981361
NCBI ET61	2020	NA	Caecum chicken	Czech-Republic	R	Illumina	130	4412199	62609	19	28.60	98.27	0	3932	57	8	1	SAMN34359474
NCBI Avi11	2020	NA	Caecum chicken	Czech-Republic	R	Illumina	92	4475231	121723	12	28.56	98.27	0	4000	66	8	1	SAMN34359439
NCBI DKU-11	2013	NA	Human infant faeces	South-Korea	R	Illumina	2	4630814	3861517	1	28.74	98.27	0	4139	88	33		SAMN35992232
NCBI MCC 0233	2012	NA	Commercial Preparation	India	R	Illumina	3	4623548	3821098	1	28.74	98.27	0	4064	87	36	1	SAMN36287673
NCBI CBM588	1933	NA	Human stool	Japan	R	Illumina	3	4609089	3806640	1	28.72	98.27	0	4065	88	30	1	SAMN36905461
NCBI CLA-SR-H018	2022	NA	Human stool	Germany	R	Illumina	62	4503563	180604	8	28.50	98.27	0	3960	58	6		SAMN40631392
NCBI YIM B08220	2021	NA	Plant root	China	R	Illumina	74	4372922	126266	13	28.51	98.27	0	3898	33	7	1	SAMN42503922
NCBI YIM B08221	2021	NA	Plant root	China	R	Illumina	124	4359221	68815	22	28.54	98.27	0	3888	35	7	1	SAMN42503922
NCBI YIM B08182	2021	NA	Plant root	China	R	Illumina	82	4367616	104082	15	28.50	98.27	0	3894	33	4	1	SAMN42503922
NCBI YIM B08209	2021	NA	Plant root	China	R	Illumina	100	4365343	99367	16	28.51	98.27	0	3892	28	6	1	SAMN42503922
NCBI YIM B08212	2021	NA	Plant root	China	R	Illumina	92	4632260	130839	12	28.49	98.27	0	4168	34	4	1	SAMN42503922
NCBI YIM B08199	2021	NA	Plant root	China	R	Illumina	74	4618386	142102	9	28.46	98.27	0	4151	41	5	1	SAMN42503922
NCBI YIM B08208	2021	NA	Plant root	China	R	Illumina	79	4616491	134084	10	28.45	98.27	0	4152	35	4	1	SAMN42503922
NCBI YIM B08210	2021	NA	Plant root	China	R	Illumina	155	4811665	52862	24	28.41	98.27	0	4450	34	3	1	SAMN42503922
NCBI YIM B08217	2021	NA	Plant root	China	R	Illumina	59	4624001	185121	8	28.46	98.27	0	4157	41	7	1	SAMN42503922
NCBI YIM B08216	2021	NA	Plant root	China	R	Illumina	58	4626531	179408	7	28.46	98.27	0	4164	34	6	1	SAMN42503922
NCBI YIM B08215	2021	NA	Plant root	China	R	Illumina	70	4615631	130839	10	28.45	98.27	0	4150	40	4	1	SAMN42503922
NCBI YIM B08200	2021	NA	Plant root	China	R	Illumina	71	4616771	142451	9	28.45	98.27	0	4157	25	4	1	SAMN42503922
NCBI YIM B08178	2021	NA	Plant root	China	R	Illumina	72	4373867	126266	13	28.51	98.27	0	3899	33	7	1	SAMN42503922
NCBI YIM B08205	2021	NA	Plant root	China	R	Illumina	78	4371213	125885	13	28.51	98.27	0	3895	36	7	1	SAMN42503922

NCBI YIM B08213	2021	NA	Plant root	China	R	Illumina	60	4627314	178931	8	28.46	98.27	0	4166	43	7	1	SAMN42503922
NCBI YIM B08186	2021	NA	Plant root	China	R	Illumina	65	4620291	151856	8	28.45	98.27	0	4156	36	4	2	SAMN42503922
NCBI YIM B08195	2021	NA	Plant root	China	R	Illumina	194	4657636	75943	18	28.66	98.27	0.47	4141	40	8	2	SAMN42503922
NCBI YIM B08179	2021	NA	Plant root	China	R	Illumina	71	4619940	150284	9	28.45	98.27	0	4155	32	4	2	SAMN42503922
NCBI YIM B08207	2021	NA	Plant root	China	R	Illumina	73	4372185	126266	13	28.51	98.27	0	3897	37	7	1	SAMN42503922
NCBI YIM B08197	2021	NA	Plant root	China	R	Illumina	63	4622853	151856	8	28.47	98.27	0	4158	39	6	1	SAMN42503922
NCBI YIM B08201	2021	NA	Plant root	China	R	Illumina	74	4622153	130839	10	28.45	98.27	0	4161	35	5	2	SAMN42503922
NCBI YIM B08203	2021	NA	Plant root	China	R	Illumina	76	4621610	149537	10	28.45	98.27	0	4159	36	5	2	SAMN42503922
NCBI YIM B08185	2021	NA	Plant root	China	R	Illumina	71	4620172	134084	10	28.45	98.27	0	4158	34	4	2	SAMN42503922
NCBI YIM B08202	2021	NA	Plant root	China	R	Illumina	81	4366721	90572	15	28.49	98.27	0	3894	33	3	2	SAMN42503922
NCBI YIM B08184	2021	NA	Plant root	China	R	Illumina	44	4538338	226287	7	28.56	98.27	0	4053	32	5	1	SAMN42503922
NCBI YIM B08163	2021	NA	Plant root	China	R	Illumina	66	4617107	178486	7	28.45	98.27	0	4154	36	6	1	SAMN42503922
NCBI YIM B08183	2021	NA	Plant root	China	R	Illumina	100	4618368	108262	13	28.46	98.27	0	4158	39	6	2	SAMN42503922
NCBI YIM B08181	2021	NA	Plant root	China	R	Illumina	75	4617264	131410	12	28.45	98.27	0	4156	38	3	2	SAMN42503922
NCBI YIM B08164	2021	NA	Plant root	China	R	Illumina	35	4541721	262402	6	28.56	98.27	0	4055	35	5	2	SAMN42503922
NCBI YIM B08153	2021	NA	Plant root	China	R	Illumina	100	4668921	137070	9	28.54	98.27	4.12	4185	36	6	1	SAMN42503922
NCBI YIM B08176	2021	NA	Plant root	China	R	Illumina	70	4374622	126266	12	28.51	98.27	0	3900	36	7	1	SAMN42503922
NCBI YIM B08175	2021	NA	Plant root	China	R	Illumina	38	4587012	247566	7	28.51	98.27	0	4077	45	7	1	SAMN42503922
NCBI YIM B08166	2021	NA	Plant root	China	R	Illumina	75	4373338	126266	13	28.51	98.27	0	3897	32	7	1	SAMN42503922
NCBI YIM B08165	2021	NA	Plant root	China	R	Illumina	75	4372743	126266	13	28.50	98.27	0	3896	35	7	1	SAMN42503922
NCBI YIM B08168	2021	NA	Plant root	China	R	Illumina	142	4358766	52679	24	28.52	98.27	0	3884	33	5	2	SAMN42503922
NCBI YIM B08159	2021	NA	Plant root	China	R	Illumina	88	4365290	89682	16	28.50	98.27	0	3889	30	6	2	SAMN42503922
NCBI YIM B08174	2021	NA	Plant root	China	R	Illumina	95	4346373	86390	17	28.50	98.27	0	3884	28	6	1	SAMN42503922
NCBI YIM B08172	2021	NA	Plant root	China	R	Illumina	90	4616869	135981	10	28.45	98.27	0	4158	29	4	2	SAMN42503922
NCBI YIM B08150	2021	NA	Plant root	China	R	Illumina	70	4373292	115053	12	28.51	98.27	0	3898	50	5	1	SAMN42503922
NCBI YIM B08173	2021	NA	Plant root	China	R	Illumina	60	4622696	126266	13	28.51	98.27	0	4155	47	6	1	SAMN42503922
NCBI YIM B08171	2021	NA	Plant root	China	R	Illumina	72	4371898	126266	13	28.51	98.27	0	3897	30	7	1	SAMN42503922
NCBI YIM B08149	2021	NA	Plant root	China	R	Illumina	72	4372699	126266	12	28.51	98.27	0	3897	31	7	1	SAMN42503922
NCBI YIM B08152	2021	NA	Plant root	China	R	Illumina	70	4616911	151856	9	28.45	98.27	0	4156	33	4	2	SAMN42503922

NCBI YIM B08158	2021	NA	Plant root	China	R	Illumina	78	4619062	137070	9	28.45	98.27	0	4154	34	5	2	SAMN42503922
NCBI YIM B08156	2021	NA	Plant root	China	R	Illumina	68	4618107	135981	9	28.44	98.27	0	4155	33	3	2	SAMN42503922
NCBI YIM B08154	2021	NA	Plant root	China	R	Illumina	38	4538889	226287	8	28.55	98.27	0	4057	34	3	1	SAMN42503922
NCBI YIM B08147	2021	NA	Plant root	China	R	Illumina	36	4540180	262402	6	28.56	98.27	0	4055	34	4	1	SAMN42503922
NCBI YIM B08155	2021	NA	Plant root	China	R	Illumina	71	4614918	137070	9	28.44	98.27	0	4151	34	3	2	SAMN42503922
NCBI YIM B08144	2021	NA	Plant root	China	R	Illumina	71	4371805	126266	12	28.51	98.27	0	3897	38	6	1	SAMN42503922
NCBI YIM B08036	2021	NA	Plant root	China	R	Illumina	34	4546395	354434	5	28.57	98.27	0	4058	47	5	1	SAMN42503922
NCBI YIM B08143	2021	NA	Plant root	China	R	Illumina	71	4378419	126310	13	28.51	98.27	0	3899	41	7	1	SAMN42503922
NCBI YIM B08177	2021	NA	Plant root	China	R	Illumina	73	4372375	126266	14	28.51	98.27	0	3897	34	7	1	SAMN42503922
NCBI GBW-N1	2017	NA	Tissue sample	China	R	Illumina	2	4630768	3861478	1	28.74	98.27	0	4155	88	33		SAMN43557426
NCBI UTH001	2021	NA	NA	Japan	R	Illumina	73	4498141	141956	9	28.53	98.27	0	4009	84	7	1	SAMD00631555

* 1: preterm infants with diagnosis of necrotizing enterocolitis (NEC). 0: preterm infants with no diagnosis of necrotizing enterocolitis (NEC). NA: not applicable.

Table S2. Alignment scores of *C. butyricum* isolates obtained with Snippy.

ID	LENGTH	ALIGNED	UNALIGNED	VARIANT	HET	MASKED	LOWCOV
**CB100**	4698320	3895675	733247	29024	306	0	69092
**CB101**	4698320	3906544	727679	28883	306	0	63791
**CB102**	4698320	3875099	738562	28552	313	0	84346
**CB103**	4698320	3947819	675347	27197	401	0	74753
**CB104**	4698320	3896505	731592	27027	314	0	69909
**CB105**	4698320	3925853	704290	26674	209	0	67968
**CB106**	4698320	3881054	728827	24325	566	0	87873
**CB107**	4698320	3881138	724824	24012	520	0	91838
**CB108**	4698320	3943107	670394	25683	985	0	83834
**CB109**	4698320	3967488	662163	25536	652	0	68017
**CB10**	4698320	3970240	656914	25615	531	0	70635
**CB110**	4698320	3933064	700595	22979	283	0	64378
**CB111**	4698320	3945678	691681	22989	252	0	60709
**CB112**	4698320	3926680	697853	23055	576	0	73211
**CB113**	4698320	3944479	692713	22948	259	0	60869
**CB114**	4698320	3928874	698301	23045	663	0	70482
**CB115**	4698320	3940845	697676	22928	290	0	59509
**CB116**	4698320	3943189	696741	22916	315	0	58075
**CB117**	4698320	3947899	679160	25344	712	0	70549
**CB118**	4698320	3932374	703265	22639	130	0	62551
**CB119**	4698320	3948535	679639	25123	711	0	69435
**CB11**	4698320	3969528	655911	25103	577	0	72304
**CB120**	4698320	3889748	728198	22441	123	0	80251
**CB121**	4698320	3941673	698037	22514	288	0	58322
**CB122**	4698320	3943200	696485	22520	293	0	58342
**CB123**	4698320	3942671	696352	22518	295	0	59002

**CB124**	4698320	3942716	696935	22534	258	0	58411
**CB125**	4698320	3815229	781892	56335	276	0	100923
**CB126**	4698320	3834550	774574	56230	342	0	88854
**CB127**	4698320	3963141	662700	24212	1191	0	71288
**CB128**	4698320	3932115	705254	21785	218	0	60733
**CB129**	4698320	3944481	694035	21793	246	0	59558
**CB12**	4698320	3848681	747814	54999	244	0	101581
**CB130**	4698320	3942274	695036	21590	229	0	60781
**CB131**	4698320	4000707	633892	21480	163	0	63558
**CB132**	4698320	3936510	699860	21361	301	0	61649
**CB133**	4698320	3935247	704379	21303	264	0	58430
**CB134**	4698320	3988484	643327	21380	142	0	66367
**CB135**	4698320	3900578	719665	21196	791	0	77286
**CB136**	4698320	3927134	695253	21070	647	0	75286
**CB137**	4698320	3925479	697080	21087	599	0	75162
**CB138**	4698320	3902055	709864	20925	560	0	85841
**CB139**	4698320	3877267	729438	20759	662	0	90953
**CB13**	4698320	3944691	695554	21086	360	0	57715
**CB140**	4698320	3830891	775039	54067	324	0	92066
**CB141**	4698320	3829015	776121	54068	312	0	92872
**CB142**	4698320	3998081	640730	18624	316	0	59193
**CB143**	4698320	3931251	702619	21096	441	0	64009
**CB144**	4698320	3806905	784563	52057	302	0	106550
**CB145**	4698320	3823458	772009	52031	333	0	102520
**CB146**	4698320	3940082	698423	20050	265	0	59550
**CB147**	4698320	3927253	696716	19839	642	0	73709
**CB148**	4698320	3927450	696865	19834	616	0	73389
**CB149**	4698320	3928718	697568	19835	589	0	71445
**CB14**	4698320	3970769	658007	22237	526	0	69018
**CB150**	4698320	3969387	655938	22232	536	0	72459
**CB151**	4698320	3762859	844561	51191	429	0	90471
**CB152**	4698320	3956054	674088	21338	352	0	67826
**CB153**	4698320	3971351	657823	21971	577	0	68569

**CB154**	4698320	3970259	656398	21960	503	0	71160
**CB155**	4698320	3945983	690207	18768	318	0	61812
**CB156**	4698320	3962276	668857	21927	902	0	66285
**CB157**	4698320	3950793	674667	21707	649	0	72211
**CB158**	4698320	3954533	668646	21711	768	0	74373
**CB159**	4698320	3974843	651554	18869	9847	0	62076
**CB15**	4698320	3872423	764948	18438	288	0	60661
**CB160**	4698320	3831738	772317	48854	406	0	93859
**CB161**	4698320	3911733	707954	17874	2003	0	76630
**CB162**	4698320	3927497	697651	17802	571	0	72601
**CB163**	4698320	3917623	704512	17789	592	0	75593
**CB164**	4698320	3922988	698237	17715	529	0	76566
**CB165**	4698320	3926855	695196	17711	621	0	75648
**CB166**	4698320	3926828	695070	17722	644	0	75778
**CB167**	4698320	3969912	656182	20386	608	0	71618
**CB168**	4698320	3994646	638006	19695	504	0	65164
**CB169**	4698320	3971151	657303	20357	696	0	69170
**CB16**	4698320	3972176	657780	20361	269	0	68095
**CB170**	4698320	3925192	697031	17660	652	0	75445
**CB171**	4698320	3885825	735290	17920	713	0	76492
**CB172**	4698320	3969744	660663	20265	556	0	67357
**CB173**	4698320	3913720	707125	17642	493	0	76982
**CB174**	4698320	3923126	700141	17510	609	0	74444
**CB175**	4698320	3969116	658770	19388	2840	0	67594
**CB176**	4698320	3944725	697012	17388	409	0	56174
**CB177**	4698320	3959247	665449	19421	674	0	72950
**CB178**	4698320	3954798	668785	19553	683	0	74054
**CB179**	4698320	3945817	679233	19454	623	0	72647
**CB17**	4698320	3895905	734769	18320	261	0	67385
**CB180**	4698320	3931628	702644	17246	594	0	63454
**CB181**	4698320	3920748	702280	16826	854	0	74438
**CB182**	4698320	3969016	660100	19092	612	0	68592
**CB183**	4698320	3944951	698010	17077	394	0	54965

**CB184**	4698320	3968718	657793	19046	536	0	71273
**CB185**	4698320	3945078	691508	16980	278	0	61456
**CB186**	4698320	3926757	694858	16623	573	0	76132
**CB187**	4698320	3928256	697231	16631	724	0	72109
**CB188**	4698320	3933111	700969	17414	284	0	63956
**CB189**	4698320	3911898	721014	18308	512	0	64896
**CB18**	4698320	3890157	739492	18152	295	0	68376
**CB190**	4698320	3936309	702136	17388	321	0	59554
**CB191**	4698320	3940786	698592	17021	295	0	58647
**CB192**	4698320	3944152	695542	16962	313	0	58313
**CB193**	4698320	3903526	719546	17653	275	0	74973
**CB194**	4698320	3924018	704648	17652	310	0	69344
**CB195**	4698320	3851431	774677	17699	162	0	72050
**CB196**	4698320	3904920	718326	17060	255	0	74819
**CB197**	4698320	3835276	786567	16735	274	0	76203
**CB198**	4698320	3829090	784501	16637	255	0	84474
**CB199**	4698320	3915001	711725	16581	255	0	71339
**CB19**	4698320	3900962	726373	16934	255	0	70730
**CB1**	4698320	3969097	655689	17590	547	0	72987
**CB200**	4698320	3823387	788914	16912	241	0	85778
**CB20**	4698320	3920154	709274	16776	266	0	68626
**CB21**	4698320	3878949	745946	16841	209	0	73216
**CB22**	4698320	4027783	605397	14197	386	0	64754
**CB23**	4698320	3883364	741920	16831	206	0	72830
**CB24**	4698320	3916380	708182	16669	383	0	73375
**CB25**	4698320	3863103	765972	16954	392	0	68853
**CB26**	4698320	3913770	707800	16968	608	0	76142
**CB27**	4698320	3821532	800589	16719	277	0	75922
**CB28**	4698320	3897108	718566	16913	583	0	82063
**CB29**	4698320	3881264	737288	16298	341	0	79427
**CB2**	4698320	3969434	656281	16784	602	0	72003
**CB30**	4698320	3924618	706072	16189	240	0	67390
**CB31**	4698320	3908943	731186	14961	526	0	57665

**CB32**	4698320	3898316	736626	15994	322	0	63056
**CB33**	4698320	3791478	806719	41647	300	0	99823
**CB34**	4698320	3846620	779508	15622	205	0	71987
**CB35**	4698320	3596200	1003992	39550	229	0	97899
**CB36**	4698320	3873624	752585	14943	236	0	71875
**CB37**	4698320	3920136	716639	14240	597	0	60948
**CB38**	4698320	3793808	802094	39503	339	0	102079
**CB39**	4698320	3795297	802017	39492	304	0	100702
**CB3**	4698320	3971280	657680	16027	562	0	68798
**CB40**	4698320	3900980	730430	15251	335	0	66575
**CB41**	4698320	3905844	728741	15232	308	0	63427
**CB42**	4698320	3906075	728255	15235	303	0	63687
**CB43**	4698320	3902574	731902	15269	307	0	63537
**CB44**	4698320	3683882	893561	38958	367	0	120510
**CB45**	4698320	3848860	763674	14372	395	0	85391
**CB46**	4698320	3717016	864097	37993	430	0	116777
**CB47**	4698320	3700929	890827	37961	529	0	106035
**CB48**	4698320	3504730	1084440	36901	496	0	108654
**CB49**	4698320	3725644	861378	36881	435	0	110863
**CB4**	4698320	3970089	657090	14768	515	0	70626
**CB50**	4698320	3893839	725026	13650	409	0	79046
**CB51**	4698320	3620476	958652	36398	435	0	118757
**CB52**	4698320	3752520	829301	36220	530	0	115969
**CB53**	4698320	3650515	924268	35986	311	0	123226
**CB54**	4698320	3807838	809205	13344	161	0	81116
**CB55**	4698320	3879702	745758	12948	359	0	72501
**CB56**	4698320	3725936	865619	35628	305	0	106460
**CB57**	4698320	3689387	885084	35411	303	0	123546
**CB58**	4698320	3709457	870517	35259	352	0	117994
**CB59**	4698320	3725988	854919	35032	381	0	117032
**CB5**	4698320	3971314	657666	14002	579	0	68761
**CB60**	4698320	3775993	818013	35019	316	0	103998
**CB61**	4698320	3931253	706603	13239	377	0	60087

**CB62**	4698320	3930908	707244	13246	354	0	59814
**CB63**	4698320	3899296	736048	13325	340	0	62636
**CB64**	4698320	3923911	712269	13237	247	0	61893
**CB65**	4698320	3896522	725863	13202	267	0	75668
**CB66**	4698320	3890446	731988	13130	332	0	75554
**CB67**	4698320	3879699	755417	13877	1526	0	61678
**CB68**	4698320	3895361	735590	13015	450	0	66919
**CB69**	4698320	3781977	813808	34201	364	0	102171
**CB6**	4698320	3971947	657862	13630	610	0	67901
**CB70**	4698320	3903798	732137	13018	304	0	62081
**CB71**	4698320	3904977	730405	13023	313	0	62625
**CB72**	4698320	3699920	870179	33861	277	0	127944
**CB73**	4698320	3795218	801626	33865	377	0	101099
**CB74**	4698320	3794171	804654	33857	370	0	99125
**CB75**	4698320	3906061	728232	12914	292	0	63735
**CB76**	4698320	3906484	728343	12918	331	0	63162
**CB77**	4698320	3891472	735148	12907	319	0	71381
**CB78**	4698320	3880899	743691	12915	303	0	73427
**CB79**	4698320	3886561	736545	12897	307	0	74907
**CB7**	4698320	3967839	656592	13414	575	0	73314
**CB80**	4698320	3795782	799494	33684	352	0	102692
**CB81**	4698320	3922799	712378	11898	556	0	62587
**CB82**	4698320	3793790	801124	33681	367	0	103039
**CB83**	4698320	3776016	808199	33649	310	0	113795
**CB84**	4698320	3797361	798698	33627	475	0	101786
**CB85**	4698320	3901367	729632	12874	349	0	66972
**CB86**	4698320	3856329	770099	12963	165	0	71727
**CB87**	4698320	4020745	616238	10831	989	0	60348
**CB88**	4698320	3904944	728250	12848	1512	0	63614
**CB89**	4698320	3906819	725790	12843	416	0	65295
**CB8**	4698320	3969546	656270	13387	597	0	71907
**CB90**	4698320	3905755	728801	12851	338	0	63426
**CB91**	4698320	3900317	732331	12839	374	0	65298

**CB92**	4698320	3950976	684071	10813	961	0	62312
**CB93**	4698320	4034744	596647	10153	7189	0	59740
**CB94**	4698320	3871531	742563	11836	952	0	83274
**CB95**	4698320	3899123	731836	11822	325	0	67036
**CB96**	4698320	3894036	733317	11829	333	0	70634
**CB97**	4698320	3856869	737928	11860	308	0	103215
**CB98**	4698320	3903977	728377	11765	335	0	65631
**CB99**	4698320	3893842	733732	11755	309	0	70437
**CB9**	4698320	3927954	696515	10934	777	0	73074
**NCBI BoNT E BL5262**	4698320	3767475	844574	20583	211	0	86060
**NCBI HM-68**	4698320	3940657	693573	11370	367	0	63723
**NCBI KNU-L09**	4698320	3951320	676730	12505	627	0	69643
**NCBI JKY6D1**	4698320	3935914	689916	12503	987	0	71503
**NCBI TOA**	4698320	3953288	673303	12447	469	0	71260
**NCBI 29-1**	4698320	3948995	677007	12420	515	0	71803
**NCBI 4-1**	4698320	3942730	691433	11325	361	0	63796
**NCBI NBRC 13949**	4698320	4552939	127171	7	11	0	18199
**NCBI NBRC 3315**	4698320	3934930	701080	11323	295	0	62015
**NCBI NBRC 13949**	4698320	4526481	149717	5	17	0	22105
**NCBI CFSA3987**	4698320	4029696	601086	11913	901	0	66637
**NCBI CFSA3989**	4698320	4029622	601450	11904	895	0	66353
**NCBI H102020561**	4698320	4017778	608070	9804	779	0	71693
**NCBI H102020560**	4698320	4020472	607728	9771	761	0	69359
**NCBI ATCC 43755**	4698320	3624849	976981	19912	244	0	96246
**NCBI BL-5262-9RE**	4698320	3762570	844093	19913	209	0	91448
**NCBI 16-3**	4698320	3942360	690366	11111	352	0	65242
**NCBI DJ064**	4698320	3942916	690850	11113	367	0	64187
**NCBI HYCB**	4698320	3955810	673223	12195	572	0	68715
**NCBI DJ046**	4698320	3944731	691556	11099	448	0	61585
**NCBI DJ075**	4698320	3942367	690259	11104	322	0	65372
**NCBI DJ013**	4698320	3934750	700138	11100	340	0	63092
**NCBI CBUT**	4698320	3803480	823494	12517	314	0	71032
**NCBI CFSA-TJ-E**	4698320	4035708	599600	9469	836	0	62176

**NCBI LV1**	4698320	3943254	691581	11071	329	0	63156
**NCBI MALS002**	4698320	3954209	686101	10251	4330	0	53680
**NCBI ET61**	4698320	3855881	766069	11564	281	0	76089
**NCBI Avi11**	4698320	3918097	710116	11500	453	0	69654
**NCBI DKU-11**	4698320	3942613	690181	10326	337	0	65189
**NCBI MCC 0233**	4698320	3958810	670102	11404	562	0	68846
**NCBI CBM588**	4698320	3957859	667871	11404	527	0	72063
**NCBI CLA-SR-H018**	4698320	3831988	765863	30009	467	0	100002
**NCBI YIM B08220**	4698320	3636706	960227	24488	571	0	100816
**NCBI YIM B08221**	4698320	3619647	970430	24444	562	0	107681
**NCBI YIM B08182**	4698320	3634223	962221	24440	552	0	101324
**NCBI YIM B08209**	4698320	3629147	965058	24448	525	0	103590
**NCBI YIM B08212**	4698320	3939338	666545	24207	671	0	91766
**NCBI YIM B08199**	4698320	3943736	662826	24205	599	0	91159
**NCBI YIM B08208**	4698320	3940850	665360	24193	617	0	91493
**NCBI YIM B08210**	4698320	3916299	714960	10018	215	0	66846
**NCBI YIM B08217**	4698320	3946888	661780	24184	588	0	89064
**NCBI YIM B08216**	4698320	3946105	662376	24180	577	0	89262
**NCBI YIM B08215**	4698320	3943208	664834	24183	631	0	89647
**NCBI YIM B08200**	4698320	3941583	665000	24191	595	0	91142
**NCBI YIM B08178**	4698320	3637024	959799	24388	573	0	100924
**NCBI YIM B08205**	4698320	3636014	960966	24388	557	0	100783
**NCBI YIM B08213**	4698320	3947253	661084	24178	562	0	89421
**NCBI YIM B08186**	4698320	3944451	662912	24178	615	0	90342
**NCBI YIM B08195**	4698320	3929876	671412	24141	478	0	96554
**NCBI YIM B08179**	4698320	3942358	663900	24139	670	0	91392
**NCBI YIM B08207**	4698320	3637340	960052	24348	584	0	100344
**NCBI YIM B08197**	4698320	3944378	663599	24135	625	0	89718
**NCBI YIM B08201**	4698320	3943536	663733	24148	597	0	90454
**NCBI YIM B08203**	4698320	3941378	664609	24134	578	0	91755
**NCBI YIM B08185**	4698320	3941667	664581	24132	623	0	91449
**NCBI YIM B08202**	4698320	3634449	962372	24350	378	0	101121
**NCBI YIM B08184**	4698320	3920573	680486	27815	471	0	96790

**NCBI YIM B08163**	4698320	3945095	662957	24112	605	0	89663
**NCBI YIM B08183**	4698320	3937353	666230	24110	615	0	94122
**NCBI YIM B08181**	4698320	3940279	665213	24094	622	0	92206
**NCBI YIM B08164**	4698320	3922843	680346	27807	536	0	94595
**NCBI YIM B08153**	4698320	3942447	665305	24110	575	0	89993
**NCBI YIM B08176**	4698320	3637479	960143	24318	570	0	100128
**NCBI YIM B08175**	4698320	3917399	683676	28313	540	0	96705
**NCBI YIM B08166**	4698320	3636149	960751	24294	599	0	100821
**NCBI YIM B08165**	4698320	3635688	960965	24299	586	0	101081
**NCBI YIM B08168**	4698320	3620533	967961	24299	436	0	109390
**NCBI YIM B08159**	4698320	3630317	965890	24279	534	0	101579
**NCBI YIM B08174**	4698320	3612545	983321	24179	516	0	101938
**NCBI YIM B08172**	4698320	3939520	666202	24006	586	0	92012
**NCBI YIM B08150**	4698320	3637569	959548	24184	374	0	100829
**NCBI YIM B08173**	4698320	3946130	662111	23993	623	0	89456
**NCBI YIM B08171**	4698320	3636713	960206	24179	391	0	101010
**NCBI YIM B08149**	4698320	3636829	960322	24184	388	0	100781
**NCBI YIM B08152**	4698320	3943644	664019	23994	548	0	90109
**NCBI YIM B08158**	4698320	3942512	664880	23997	611	0	90317
**NCBI YIM B08156**	4698320	3943913	662628	23994	600	0	91179
**NCBI YIM B08154**	4698320	3922361	680630	27675	524	0	94805
**NCBI YIM B08147**	4698320	3921882	680836	27672	518	0	95084
**NCBI YIM B08155**	4698320	3943160	664259	23993	605	0	90296
**NCBI YIM B08144**	4698320	3636326	961077	24185	573	0	100344
**NCBI YIM B08036**	4698320	3924692	678260	27678	584	0	94784
**NCBI YIM B08143**	4698320	3637469	959726	24188	642	0	100483
**NCBI YIM B08177**	4698320	3636773	960275	24175	427	0	100845
**NCBI GBW-N1**	4698320	3943281	691300	9851	332	0	63407
**NCBI UTH001**	4698320	3951522	677460	10766	505	0	68833
**NCBI 2477**	4698320	3985886	654612	9368	344	0	57478
**NCBI 2478**	4698320	3925540	717543	9080	470	0	54767
**NCBI 34471**	4698320	3870134	762520	10313	213	0	65453
**NCBI 4218**	4698320	4552161	127674	6	0	0	18485

**NCBI 47601**	4698320	3937685	693158	8978	240	0	67237
**NCBI 5521**	4698320	3585370	1015896	17405	317	0	96737
**NCBI 60E3**	4698320	3585372	1015894	17405	317	0	96737
**NCBI AGR2140**	4698320	3782743	821210	27873	284	0	94083
**NCBI CDC_51208**	4698320	3745325	866108	19714	165	0	86722
**NCBI DKU-01**	4698320	3896905	724619	12033	720	0	76076
**DSMZ10702T (Reference)**	4698320	4698320	0	0	0	0	0
