# Supplementary material for: Whole-genome sequencing-based typing methods for Clostridium butyricum strains from clinical, animal, plant, and environmental sources
Source: Microbiol Spectr. 2025 Dec 12;14(2):e02619-25. doi: 10.1128/spectrum.02619-25 (PMC12889113; doi:10.1128/spectrum.02619-25)
Supplement: Supplemental figures — Figures S1 to S6. [file spectrum.02619-25-s0001.pdf]

Figure S1. Heatmap representing the degree of genome similarity based on ANI. The heatmap was derived from the high similarity (dark red) and low similarity (blue) of CDSs derived from the *C. butyricum* genomes.

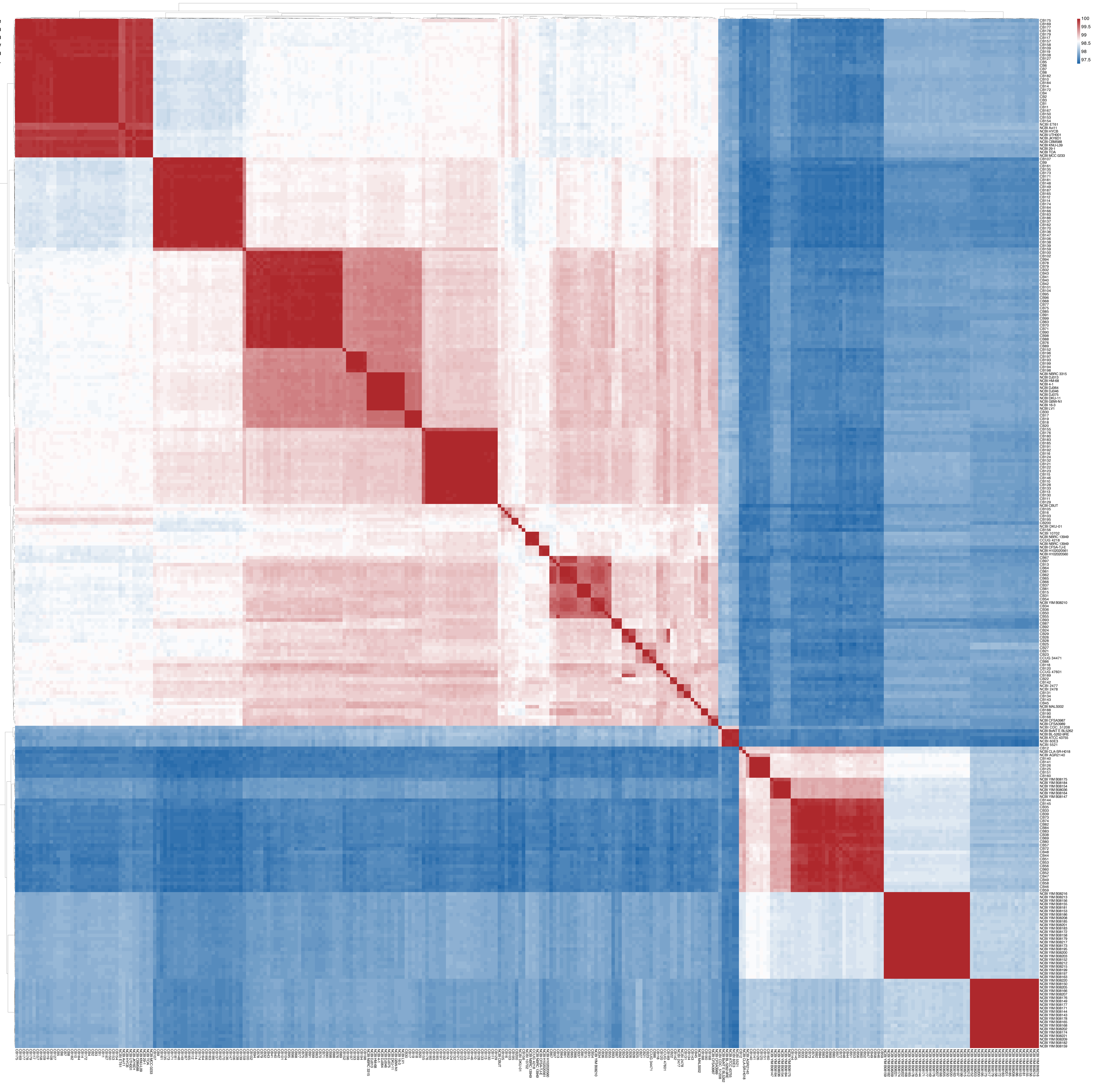

Figure S2. Minimum spanning tree comparing core-genome allelic profiles with maximum branch collapse, allowing the observation of a specific clade formed by botulinum neurotoxin type E-producing *C. butyricum* strains. NCBI_BoNT E BL5262 and NCBI BL-5262-9RE genome sequences are from the same strain.

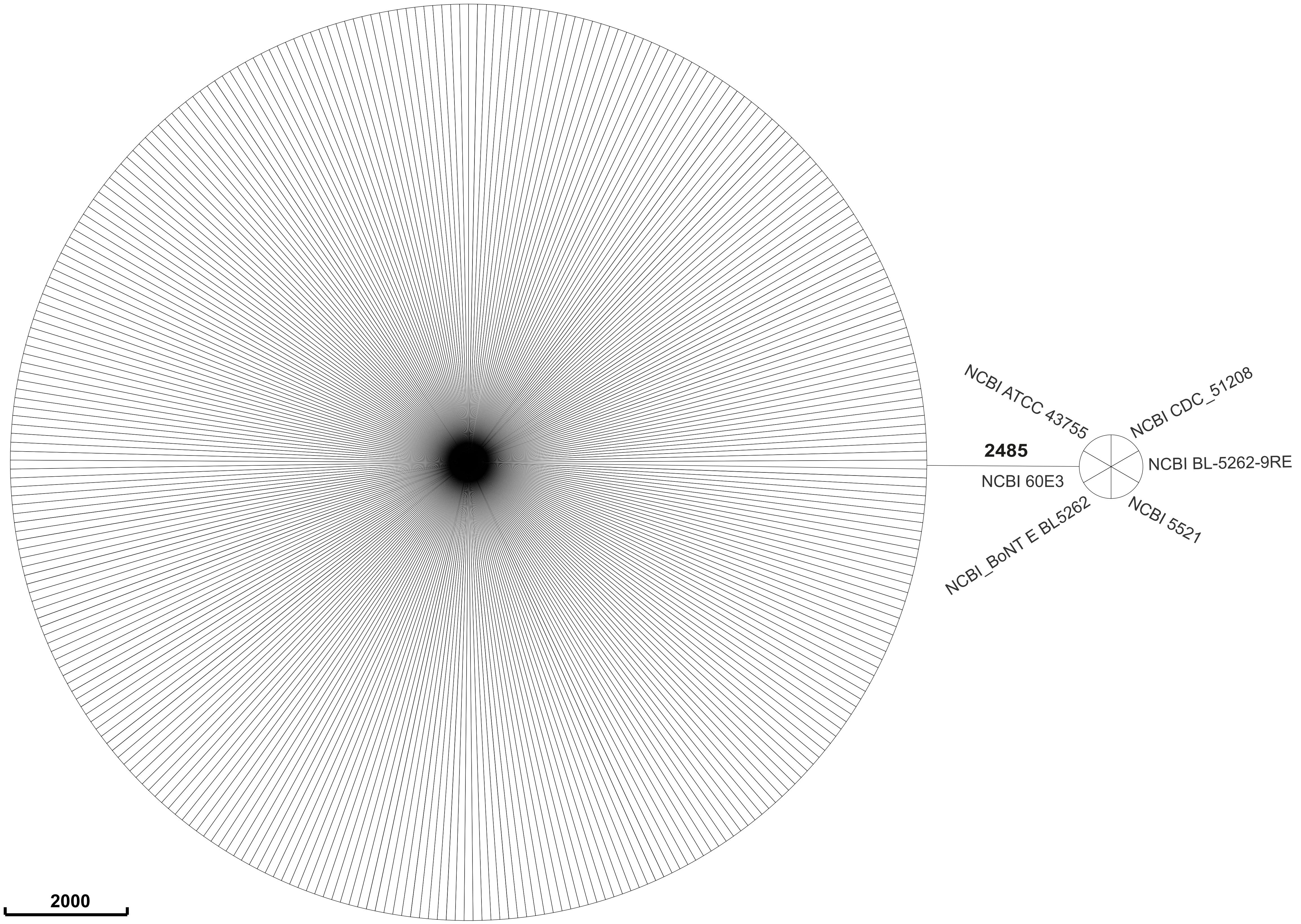

Figure S3. Minimum spanning tree based on core genome allelic profiles, colored by country of isolation. Node size corresponds to the number of strains represented. The numbers on the connecting lines illustrate the numbers of different alleles between connected samples.

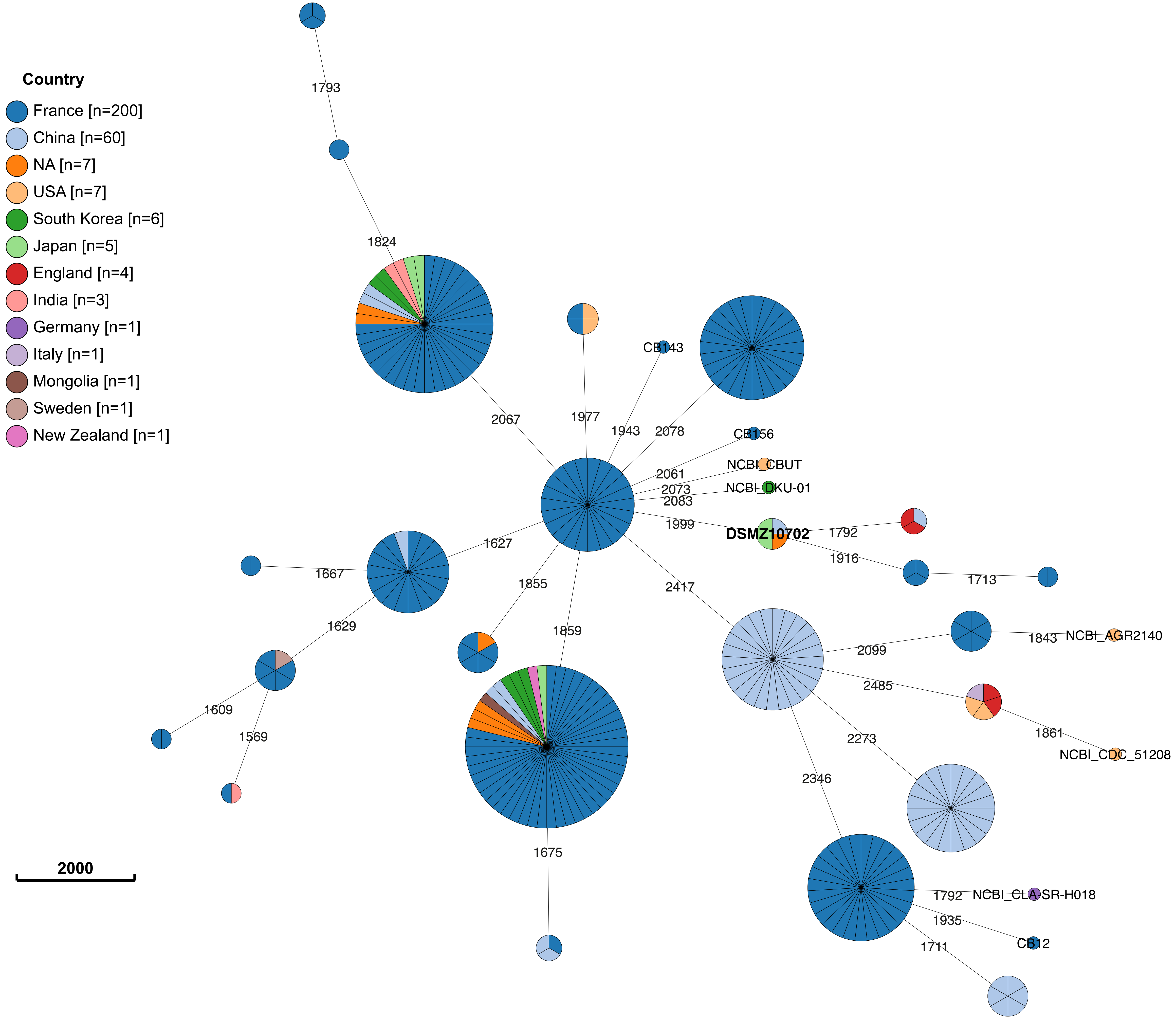

Figure S4. Minimum spanning tree based on core genome allelic profiles, colored by year of isolation. Node size corresponds to the number of strains represented. The numbers on the connecting lines illustrate the numbers of different alleles between connected samples.

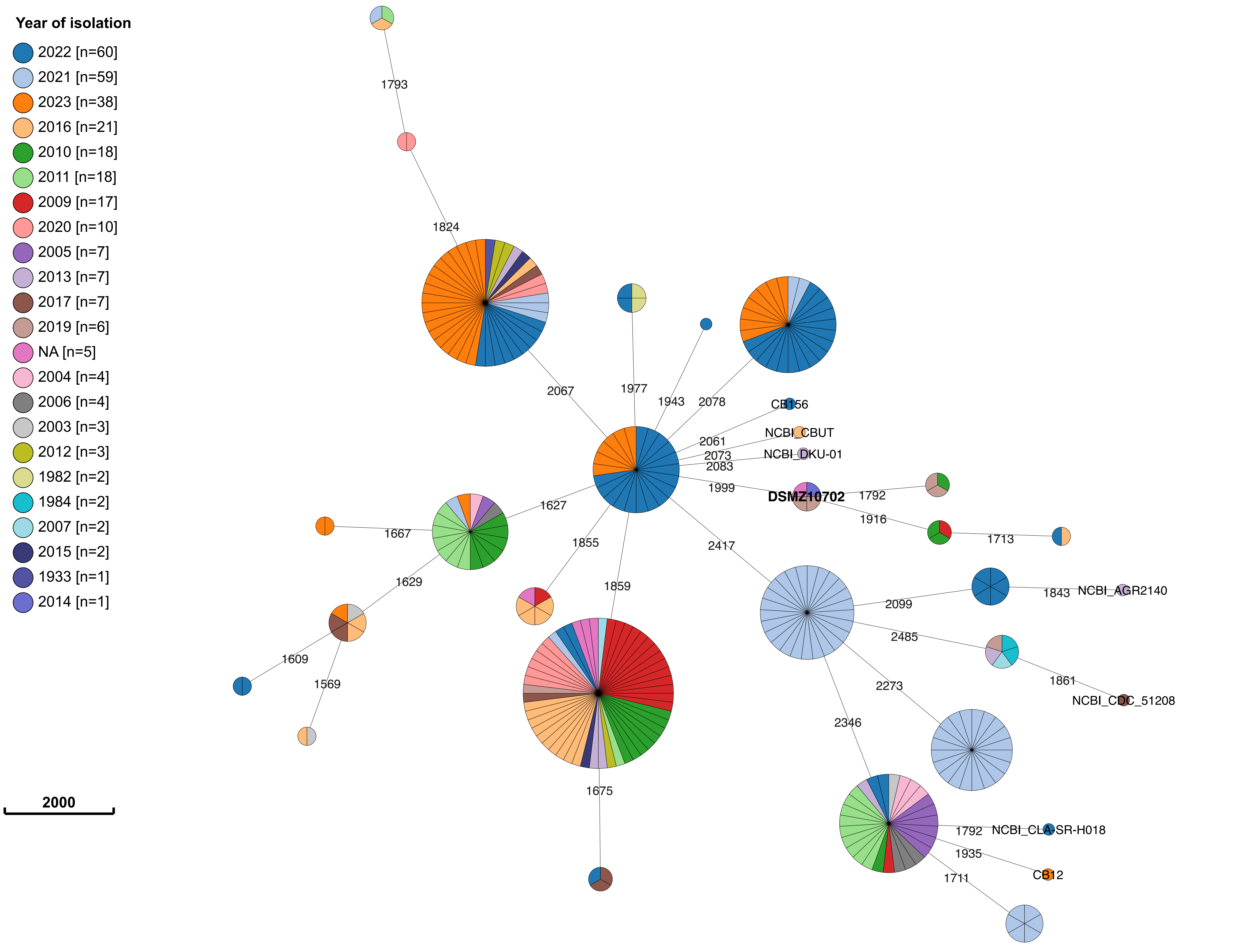

Figure S5. Minimum spanning tree based on core genome allelic profiles, colored by source of isolation. Node size corresponds to the number of strains represented. The numbers on the connecting lines illustrate the numbers of different alleles between connected samples.

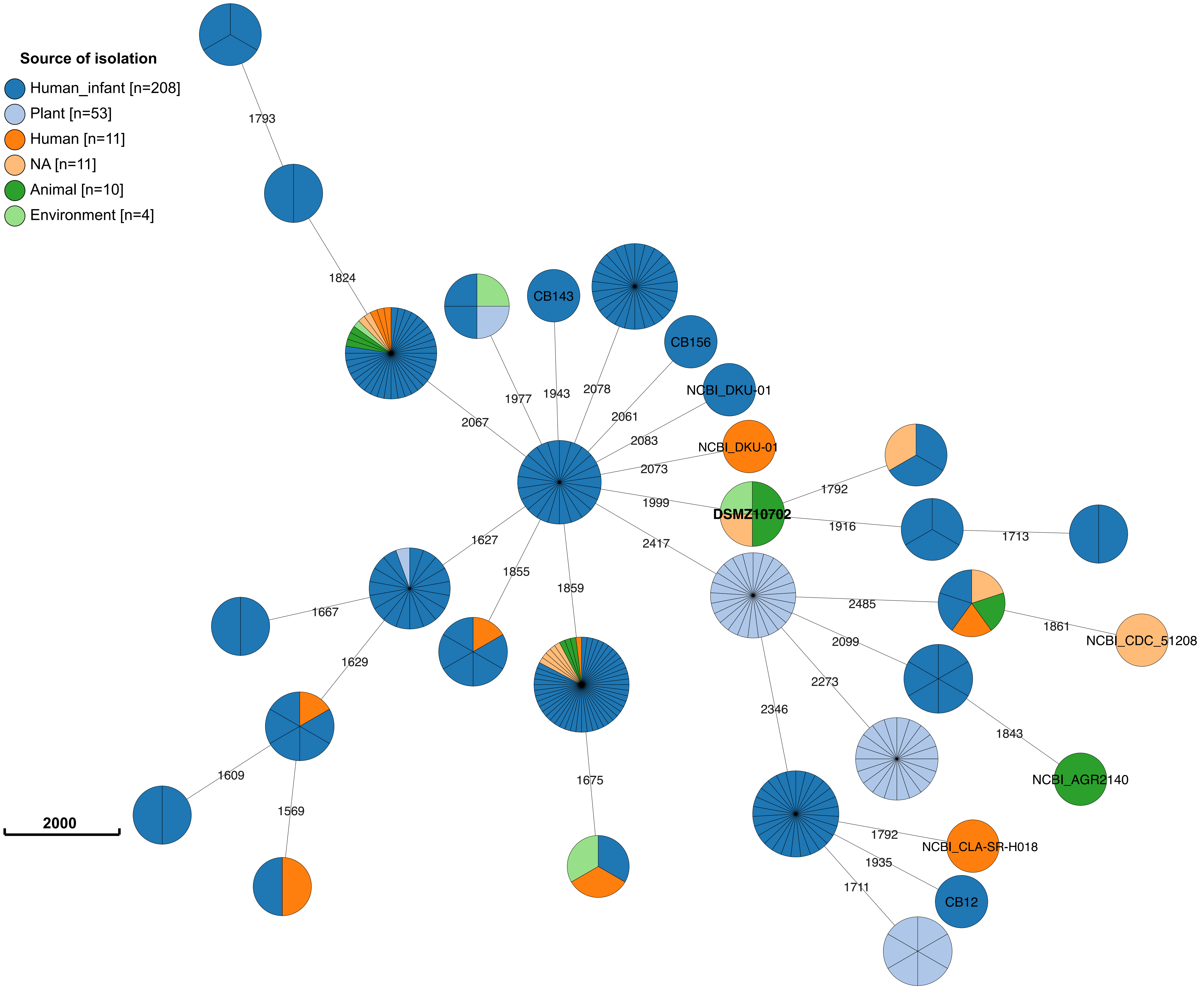

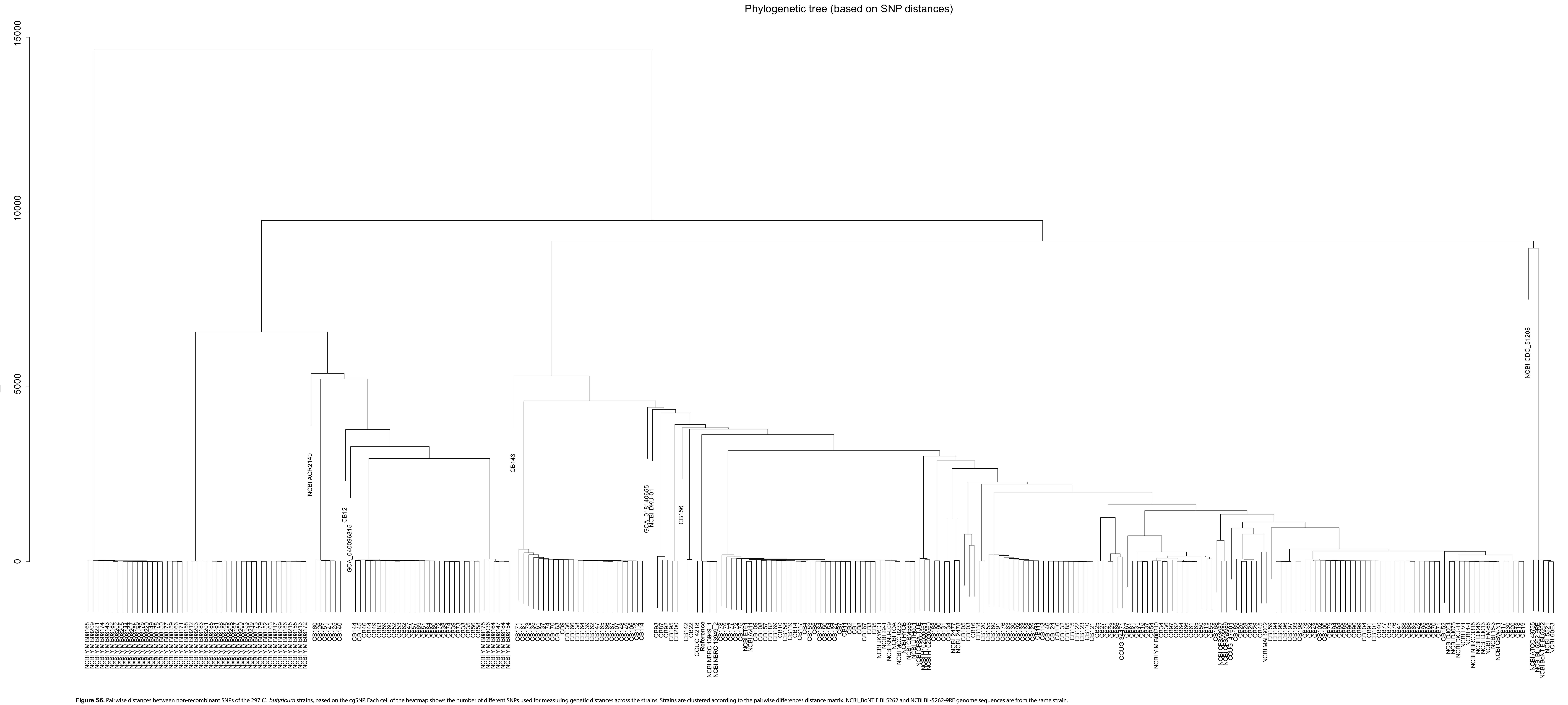

Figure S6. Pairwise distances between non-recombinant SNPs of the 297 *C. butyricum* strains, based on the cgSNP. Each cell of the heatmap shows the number of different SNPs used for measuring genetic distances across the strains. Strains are clustered according to the pairwise differences distance matrix. NCBI_BoNT E BL5262 and NCBI BL-5262-9RE genome sequences are from the same strain.
